# Evolution of Hominin Polyunsaturated Fatty Acid Metabolism: From Africa to the New World

**DOI:** 10.1093/gbe/evz071

**Published:** 2019-04-03

**Authors:** Daniel N Harris, Ingo Ruczinski, Lisa R Yanek, Lewis C Becker, Diane M Becker, Heinner Guio, Tao Cui, Floyd H Chilton, Rasika A Mathias, Timothy D O’Connor

**Affiliations:** 1 Institute for Genome Sciences, University of Maryland School of Medicine, Baltimore, Maryland; 2 Department of Medicine, University of Maryland School of Medicine, Baltimore, Maryland; 3 Program in Personalized and Genomic Medicine, University of Maryland School of Medicine, Baltimore, Maryland; 4 Department of Biostatistics, Johns Hopkins Bloomberg School of Public Health, Baltimore, Maryland; 5 GeneSTAR Research Program, Johns Hopkins University School of Medicine, Baltimore, Maryland; 6 Laboratorio de Biología Molecular, Instituto Nacional de Salud, Lima, Perú; 7 Department of Urology, Wake Forest School of Medicine, Winston-Salem, North Carolina; 8 Department of Nutritional Sciences, University of Arizona, Tucson, Arizona

**Keywords:** evolution, ancient DNA, population genetics, polyunsaturated fatty acids

## Abstract

The metabolic conversion of dietary omega-3 and omega-6 18 carbon (18C) to long chain (>20 carbon) polyunsaturated fatty acids (LC-PUFAs) is vital for human life. The rate-limiting steps of this process are catalyzed by fatty acid desaturase (*FADS*) 1 and 2. Therefore, understanding the evolutionary history of the *FADS* genes is essential to our understanding of hominin evolution. The *FADS* genes have two haplogroups, ancestral and derived, with the derived haplogroup being associated with more efficient LC-PUFA biosynthesis than the ancestral haplogroup. In addition, there is a complex global distribution of these haplogroups that is suggestive of Neanderthal introgression. We confirm that Native American ancestry is nearly fixed for the ancestral haplogroup, and replicate a positive selection signal in Native Americans. This positive selection potentially continued after the founding of the Americas, although simulations suggest that the timing is dependent on the allele frequency of the ancestral Beringian population. We also find that the Neanderthal *FADS* haplotype is more closely related to the derived haplogroup and the Denisovan clusters closer to the ancestral haplogroup. Furthermore, the derived haplogroup has a time to the most recent common ancestor of 688,474 years before present. These results support an ancient polymorphism, as opposed to Neanderthal introgression, forming in the *FADS* region during the Pleistocene with possibly differential selection pressures on both haplogroups. The near fixation of the ancestral haplogroup in Native American ancestry calls for future studies to explore the potential health risk of associated low LC-PUFA levels in these populations.

## Introduction

The metabolic conversion of omega-3 (*n*-3) and omega-6 (*n*-6) dietary 18 carbon (18C) polyunsaturated fatty acids (PUFAs) to biologically active long-chain PUFAs (>20 carbon, LC-PUFAs) is essential for human life. LC-PUFAs and their metabolites are vital structural and signaling components for numerous biological systems including brain development and function, innate immunity, and energy homeostasis (Marszalek and Lodish 2005; Calder 2013). Consequently, the capacity of populations to adapt to their PUFA environments and synthesize or ingest LC-PUFAs is an important factor to their survival (Mead et al. 1953; Steinberg et al. 1956; Alessandri et al. 2004; Kitajka et al. 2004).

In the modern western diet, the majority (>90%) of all PUFAs consumed are two plant sourced 18C-PUFAs, α-linolenic acid (18: 3*n*-3) and linoleic acid (18: 2*n*-6). Over the past 50 years, the ingestion of linoleic acid dramatically increased (∼3-fold, 6–8% of daily energy consumed) due to the addition of vegetable oil products (e.g., soybean, corn, and canola oils; and margarine/shortenings) to the modern western diet (Chilton et al. 2014). Once ingested, *n*-6 and *n*-3 18C-PUFAs can be converted into several LC-PUFAs including eicosapentaenoic acid (20: 5*n*-3), docosapentaenoic acid (22: 5*n*-3), docosahexaenoic acid (22: 6*n*-3), and arachidonic acid (20: 4*n*-6) utilizing desaturase and elongase enzymes (Alessandri et al. 2004). There are also dietary sources of preformed LC-PUFAs in eggs and certain meats containing arachidonic acid, with seafood being highly enriched for docosahexaenoic acid, eicosapentaenoic acid, and docosapentaenoic acid (Horrocks and Yeo 1999; Howe et al. 2006; Chilton et al. 2014).

The fatty acid desaturase (*FADS*) region (chr11: 61,540,615–61,664,170) contains the *FADS1* and *FADS2* genes, which encode for desaturase enzymes that catalyze the rate-limiting steps in converting 18C-PUFAs into LC-PUFAs (Nakamura and Nara 2004). It was originally assumed that LC-PUFAs’ biosynthesis from 18C-PUFAs was highly inefficient and similar in all human populations (Pawlosky et al. 2001). However, numerous studies revealed that an individual’s genetic background greatly impacts both their LC-PUFA levels and metabolic efficiencies by which LC-PUFAs are formed (Mathias et al. 2011, 2012, 2014; Ameur et al. 2012; Sergeant et al. 2012). Specifically, 1) there was an ∼30% increase in the level of circulating arachidonic acid levels in African Americans compared with European Americans, and this difference was strongly associated to differences in allele frequencies at the *FADS* locus (Mathias et al. 2011; Sergeant et al. 2012); and 2) there was no difference between the effects of the variants in the *FADS* locus on circulating PUFA levels between African Americans and European Americans (Mathias et al. 2011; Sergeant et al. 2012). Taken together, these observations strongly highlight the importance of *FADS* genetic variants in LC-PUFA metabolism at an individual’s genetic level.

In their study of the *FADS* genes, Ameur et al. (2012) found two regions of high LD in Europeans, with the first block spanning *FADS1* and part of *FADS2*. The region encompassing these blocks represents two major haplogroups, derived and ancestral. The ancestral versus derived designation is defined in part on the genotype of SNP rs174537 (chr11: 61,552,680), where the ancestral haplogroup has the same genotype as other nonhuman primates (thymine) and the derived has a human-unique genotype (guanine) (Mathias et al. 2011, 2012, 2014). The derived haplogroup is associated with more efficient conversion of 18C-PUFAs into LC-PUFAs and is most common in African populations, where a sweep to near fixation occurred before the Out-of-Africa expansion of anatomically modern humans (Mathias et al. 2012; Mathieson and Mathieson 2018). Europeans and East Asian populations are polymorphic with the derived haplogroup at greater frequency. South Asian and Oceanic populations are at greater frequency for the ancestral haplogroup and Native Americans appear to be nearly fixed for the ancestral haplogroup (Mathias et al. 2011; Ameur et al. 2012).

It is confusing that Native American populations appear to be fixed for the ancestral haplogroup that is associated with lower *FADS* activities and levels of LC-PUFAs, because this would likely be detrimental compared with the derived haplogroup (Ameur et al. 2012; Mathias et al. 2012). However, previous studies only analyzed a small Native American ancestry sample size (Ameur et al. 2012; Mathias et al. 2012) that did not provide a thorough view of the *FADS* genomic architecture in Native American ancestry. Additional recent studies found evidence that the ancestral haplogroup was under positive selection in pygmy populations on Flores Island (Tucci et al. 2018), the Greenlandic Inuit (Fumagalli et al. 2015), and Native American populations (Amorim et al. 2017). Fumagalli et al. (2015) suggested that this selection pressure is due to adapting to the dietary demands of a cold weather climate, although the exact selection pressure is unknown.

In contrast, Mathieson and Mathieson (2018) demonstrated that the ancestral haplogroup likely went to fixation shortly after the Out-of-Africa expansion, and that the selection signature identified in Native Americans represents an ancient selection event. Therefore, it is clear that there is uncertainty regarding the *FADS* haplogroup in Native Americans as to whether the positive selection of the ancestral haplogroup to near fixation occurred before or after the founding of the Americas?

The derived haplogroup is under positive selection in African (Ameur et al. 2012; Mathias et al. 2012), European (Buckley et al. 2017; Mathieson and Mathieson 2018), and East Asian populations (Liu et al. 2018). These findings provide evidence that differential selection pressures that favor one *FADS* haplogroup over another may play a critical role in the capacity of populations to adapt to different environments. Additionally, the fact that African populations are nearly fixed for the derived haplogroup while Eurasian populations are polymorphic (Mathias et al. 2011, 2012; Ameur et al. 2012), presents an interesting evolutionary puzzle, that may be explained by archaic reintroduction of the ancestral haplogroup into non-African populations.

Modern humans mixed with archaic hominins such as Neanderthals and Denisovans, after migrating out of Africa. This admixture is evidenced by the presence of archaic haplotypes in non-African genomes (Meyer et al. 2012; Prufer et al. 2014; Sankararaman et al. 2014, 2016; Vernot and Akey 2014; Vernot et al. 2016). Some of these introgressed haplotypes are associated with modern human phenotypes and diseases (Mendez et al. 2012; Huerta-Sanchez et al. 2014; Simonti et al. 2016), giving emphasis to how this evolutionary history impacted modern human biology. In addition, Neanderthals and Denisovans appear to be homozygous for the *FADS* ancestral haplogroup (Ameur et al. 2012). African populations are nearly fixed for the derived haplogroup, while non-African populations are polymorphic (Mathias et al. 2011, 2012; Ameur et al. 2012). Therefore, it is possible that the derived haplogroup rose to fixation in humans prior to the Out-of-Africa expansion followed by the ancestral haplogroup being reintroduced to non-Africans through admixture with archaic hominins in Eurasia, as previously suggested by Mathieson (2015).

An alternative hypothesis suggests that the derived haplogroup began to form prior to the divergence of Neanderthals, Denisovans, and modern humans, which was followed by differential selection pressures depending on a population’s environment, along with genetic drift. There is substantial divergence between the human derived and ancestral haplogroups (Ameur et al. 2012; Mathias et al. 2012), which suggests these haplogroups are old in the human lineage. If the derived haplogroup began to form near the divergence of these three hominins, then there would be at least 550,000 years (Prufer et al. 2014) for more mutations to occur between the two haplogroups. Previous estimates for the time to the most recent common ancestor (TMRCA) of all *FADS* human haplogroups is 1.49 Ma (Mathias et al. 2012) and the TMRCA of the derived haplogroup may be as old as 433,000 Ya (Ameur et al. 2012). However, a recent analysis examining archaic haplotypes suggested that the Neanderthal and Denisovan are more closely related to a different modern human haplogroup than they are to each other (Buckley et al. 2017). Therefore, it is possible that the TMRCA of the derived haplogroup is, in fact, older than the modern-archaic hominin divergence. Further, the differential selection pressure on the ancestral and derived haplogroups (Mathias et al. 2012; Fumagalli et al. 2015; Amorim et al. 2017; Buckley et al. 2017), in addition to drift, influenced the global haplogroup frequency distribution.

To better characterize the history of the *FADS* haplogroups, we analyzed this genetic region for signs of archaic admixture or ancient development of the derived haplogroup through the use of the 1000 Genomes Project (Altshuler et al. 2015), European American and African American genomes from GeneSTAR (Mathias et al. 2011), and data from Native American ancestry individuals from the Peruvian Genome Project (Harris et al. 2018). Further, due to the importance of LC-PUFAs to human health, we placed an added focus to further illuminate the genomic architecture of this region in Native American ancestry populations.

## Materials and Methods

### Data Preparation

We jointly called all positions in the range chr11: 61,540,615–61,664,170 genome build hg19 (Altshuler et al. 2015) in 127 African American and 156 European American genomes from GeneSTAR (Mathias et al. 2011), 67 Native American and 47 mestizo (Native American-European admixed ancestry) genomes from the Peruvian Genome Project (Harris et al. 2018) and the Neanderthal (Prufer et al. 2014) and Denisovan (Meyer et al. 2012) genome alignments downloaded from the Max Planck Institute for Evolutionary Anthropology, using GATK UnifiedGenotyper (McKenna et al. 2010). We removed all invariant sites, variant calls flagged as LowQual, sites given a quality score of ≤ 20, INDELS, or triallelic single nucleotide polymorphisms (SNPs) with vcftools v0.1.11 and PLINK1.9 (Danecek et al. 2011; Chang et al. 2015; Purcell and Chang), resulting in a high-coverage genome data set with 2,122 high-quality bialleic SNPs. We then intersected the same genomic region variant calls from all 2,504 individuals from the 1000 Genomes Project (Altshuler et al. 2015) with the high-coverage data set, to create a more diverse but low-coverage data set. We removed triallelic SNPs that were not created by a strand flip and flipped the strand in the 1000 Genomes Project genomes to correct those that were created by a strand flip, with PLINK1.9 (Chang et al. 2015; Purcell and Chang). After the intersection with the 1000 Genomes Project and filters, 899 variants remained that were present in both the 1000 Genomes Project and the high-coverage data set. Some analyses used the high-coverage data set, while others used the combined low-coverage diverse data set. The following methods sections will indicate which data set was used for each analysis by referring to the data set used as high coverage or low coverage.

### Phasing and Local Ancestry Inference

The genomes in the low- and high-coverage data sets were separately phased with SHAPEIT v2.r790 (Delaneau et al. 2012), using default settings. All missing genotype positions were imputed by SHAPEIT and, for all analyses except for local ancestry, were set back to missing. Local ancestry was calculated in the low-coverage data set on Native American and mestizo genomes with <99% Native American ancestry (Harris et al. 2018), the admixed populations from the 1000 Genomes Project (Altshuler et al. 2015), and all individuals from GeneSTAR (Mathias et al. 2011), using the default setting of RFMix (Maples et al. 2013). We used Native American and mestizo individuals with ≥ 99% Native American ancestry (Harris et al. 2018) as the Native American reference, and the CEU and YRI as the European and African reference, respectively (Altshuler et al. 2015).

### GeneSTAR Relatedness Filtering

All data sources except GeneSTAR were already filtered for kinship to remove at least third degree relations (Altshuler et al. 2015; Harris et al. 2018). Pedigree information was used to remove individuals such that no closer than third degree relations remained in the African American and European American families in GeneSTAR (Mathias et al. 2011). All of the pedigree information was previously validated with genome wide array data and genetic kinship analysis (Manichaikul et al. 2010). This kinship filtering resulted in 101 African American and 128 European American individuals to yield a total of 229 unrelated GeneSTAR samples. All analyses detailed bellow that include GeneSTAR samples only include these 229 unrelated samples.

### Ancestral and Derived Haplogroup Proportion Calculations

Haplogroups were determined as either ancestral or derived based on the genotype at SNP rs174537 (chr11: 61,552,680, ancestral = thymine, derived = guanine), which is the most representative of the haplogroups within African ancestry populations (Mathias et al. 2012). Haplogroup proportions were calculated in all populations from the low-coverage data set. In addition, we binned the haplogroup proportions by the estimated local ancestry haplotypes. This step was done on individuals with (380 African, 450 European, and 216 Native American estimated haplotypes) and without (505 African, 685 European, and 386 Native American estimated haplotypes) homozygous local ancestry calls.

### Analysis for Signs of Selection on the *FADS* Haplogroup in Peruvians

We calculated the population branch statistic (PBS) to determine if the ancestral haplogroup was under selection in Native Americans relative to the CEU and the CHB (Yi et al. 2010). An additional data set was first created by taking the Peruvian Genome Project autosome genotype calls and merging them with the 1000 Genomes Project using the same merging and filtering procedure as mentioned earlier. To calculate the PBS, we used PLINK1.9 (Chang et al. 2015; Purcell and Chang) to calculate F_ST_ between Native Americans from the Peruvian Genome Project (NatAm) (Harris et al. 2018), CEU, and CHB from the 1000 Genomes Project (Altshuler et al. 2015) over the entire genome. We then computed the PBS statistic of the form:

(1) $$\mathrm{PBS}=\;\frac{T_{\mathrm{NatAm},\mathrm{CEU}}\;+\;T_{\mathrm{NatAm},\mathrm{CHB}}\;-\;T_{\mathrm{CEU},\mathrm{CHB}}}{2}$$

throughout the entire autosome, where $T_{\mathrm{NatAm},\mathrm{CEU}}$, $T_{\mathrm{NatAm},\mathrm{CHB}}$, and $T_{\mathrm{CEU},\mathrm{CHB}}$ represent the F_ST_ log-transformed time value calculated between the Native American and CEU, Native American and CHB, and CEU and CHB populations, respectively. A *Z*-score was computed for all SNPs within ±500 kb of the *FADS* gene region by comparing to the genome wide average and its SD. The *Z*-score was then converted to a *P* value with a Bonferroni correction for multiple hypothesis testing. We also calculated the PBS statistic to assess for sites under selection in the CEU and CHB populations to serve as branch lengths in comparison to sites estimated to be under selection in Native Americans.

### Selection Simulations

**Fig. 1.**
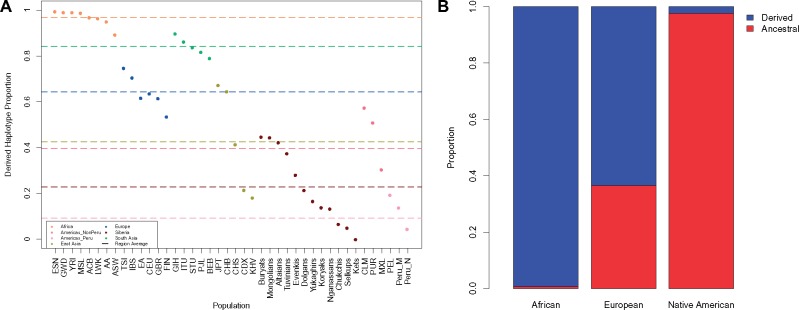
—*FADS* ancestral and derived haplogroup proportions in modern human populations. (*A*) Derived haplogroup proportion from all 1000 Genomes, Siberian, GeneSTAR African American (AA) and European American (EA) and Peruvian mestizo (Peru_M) and Native American populations (Peru_N). Dashed lines represent each geographic region’s average derived haplogroup proportion. Americas_NonPeru consists of the 1000 Genomes Native American ancestry populations and Americas_Peru represents the Peruvian Genome Project populations (Harris et al. 2018). (*B*) Ancestral (red) and derived (blue) haplogroup proportion in all local ancestry segments from the admixed 1000 Genomes Project populations, African and European Americans from GeneSTAR, and Peruvian Genome Project individuals with <99% Native American ancestry (Harris et al. 2018). (African = 505, European = 685, Native American = 386 haplotypes).

We utilized the Wright–Fisher framework to simulate the allele frequency with the following demographic factors. The simulations began with a bottleneck at the founding of the Americas (534 generations ago) (Gravel et al. 2013), which lasted 10 generations. We varied the bottleneck magnitude by reducing the effective population size to 100, 200, 300, 400, or 500 individuals. Following the bottleneck, we modeled the effective population size as an instantaneous increase to 2,000, 4,000, 6,000, 8,000, or 10,000, and kept constant for the rest of the simulation. Selection was modeled as occurring for 67, 133, 200, 267, 333, 400, 467, or 534 generations since the start of the simulation. In addition, we simulated varying forces of selection by using selection coefficients of 0, 0.1, 0.01, or 0.001, and set the codominance coefficient to 0.5. We varied the starting allele frequency between 30% and 95% with a step of 5%, consistent with observed values in East Asia and Siberia (fig. 1*A*) (Mathias et al. 2012). We then ran 100 replicates of each simulation parameter set, and calculated the proportion of replicates that resulted in the allele frequency being fixed at 100%.

### Siberian Genome Analysis

We merged genotype data from 12 Siberian populations (Rasmussen et al. 2010) with the Human Genome Diversity Panel (HGDP) (Li et al. 2008) and we removed all triallelic sites with PLINK1.9 (Chang et al. 2015; Purcell and Chang). The ancestral and derived haplogroup proportion was calculated using rs174537 frequencies. We used ADMIXTURE (Alexander et al. 2009) to calculate global ancestry proportions in all Siberian, East and South Asian populations. In addition, we included the Yoruba and French populations to serve as the African and European ancestry reference, respectively. The autosomal SNPs were first filtered by removing singletons and sites with >10% missing genotypes. We then LD pruned the data with PLINK1.9 indep-pairwise 50 5 0.5 (Chang et al. 2015; Purcell and Chang). ADMIXTURE (Alexander et al. 2009) was run on *K* 1-10, randomly 10 times for each *K*. *K* = 6 was selected as the most representative of these data due to it having the lowest cross validation. We correlated Siberian, and East and South Asian ancestral haplogroup proportions to their latitude coordinates independent of their European admixture, as determined by ADMIXTURE estimates, through computing the linear regression lm(proportion ∼ European_admixture + latitude) and only analyzed the *P* value relative to the correlation with latitude. We also computed the linear regression across all sites in the genome and divided the number of sites that have a smaller *P* value than rs174537 by the total number of sites. This resulted in an empirical *P* value to assess if rs174537 is an outlier for allele frequency correlation within Siberian genomes. Correlation to temperature would be a better comparison, although temperature data are unavailable for these regions and many of their geographic locations are remote. Therefore, we cannot use a nearby city to obtain temperature data for these populations. However, the general trend from South Asia to Siberia is a decrease in average yearly temperature (Jones, et al. 1999), which supports using latitude as an appropriate indication of temperature for this comparison.

### Ancient Humans

We downloaded SRA files from the Mota (Llorente et al. 2015), ancient Eskimo (Rasmussen et al. 2010), Anzick-1 (Rasmussen et al. 2014), MA-1 (Raghavan et al. 2014) genomes and extracted fastq files with sra-toolkit fastq-dump (Leinonen et al. 2011) to extract a sam file for the Mota individual. We downloaded bam files for all ancient European genomes (Gamba et al. 2014; Lazaridis et al. 2014; Olalde et al. 2014; Haak et al. 2015; Mathieson et al. 2015) and the Ust’Ishim individual from Siberia (Fu et al. 2014). Single read and paired-end read fastq files were generated for the Ust’Ishim individual and single read fastq files were generated for the ancient Stuttgart and Loschbour Europeans using Bedtools version 2.17.0 BAMtoFASTQ (Quinlan and Hall 2010). All fastq files were aligned to hg19 (Altshuler et al. 2015) with bwa-mem (Li 2013). The paired-end and single reads were aligned to hg19 separately with bwa-mem and then combined into one bam file using samtools v0.1.19-44428 merge (Li et al. 2009) for the Ust-Ishim individual. Duplicates were marked in the Stuttgart and Loschbour bam files with Picard Tools version 1.79 MarkDuplicates (Broad Institute). The Mota individual sam file was reformatted to be compatible with GATK (McKenna et al. 2010) and was then converted to a bam file with samtools view. All bam files were sorted and indexed with samtools (Li et al. 2009). We called all positions in the *FADS* gene region in each ancient genome individually with GATK UnifiedGenotyper (McKenna et al. 2010). Each individual was genotyped as homozygous ancestral or derived, or heterozygous based on the rs174537 genotype.

### Recombination Mapping

To determine the effectiveness of rs174537 tagging the ancestral and derived haplogroups, we used the R package rehh bifurcation.diagram function (Gautier and Vitalis 2012) to perform recombination mapping in the low-coverage data set. rs174537 was set as the variant to examine how the haplotype decayed within the *FADS* region on the ancestral or derived haplogroups due to recombination.

### Haplotype Construction and Network Analysis

We used PLINK1.9 to form LD blocks in the Native American samples with PLINK1.9’s hap command (Chang et al. 2015; Purcell and Chang). We then selected the region chr11: 61,543,499–61,591,907 with the high-coverage data set or chr11: 61,543,499–61,591,636 with the low-coverage data set to construct haplotype networks, using the R package pegas (Paradis 2010). The human–chimpanzee ancestral reconstructed reference sequence represented the outgroup haplotype (Altshuler et al. 2015). We loaded all DNA sequences into R using read.dna from the R package ape (Paradis et al. 2004), then formed the haplotypes using haplotype and a network using haploNet, both from the R package pegas (Paradis 2010).

Using the described method, we constructed haplotype networks removing all haplotypes with a count of ≤ 3 (except for the Denisovan and Neanderthal haplotypes and the human–chimpanzee ancestral reconstructed reference haplotype). Haplotype networks were then colored by ancestral versus derived, or local ancestry (if calculations available) and global population ancestry (where local ancestry calculations were unavailable). The high-coverage haplotype network also included modern human invariant sites so that sites variable in the archaic hominins and the human–chimpanzee ancestor relative to modern humans could be compared.

### Nonhominin Primate Genome Analysis

We analyzed a jointly called vcf file which contained *Gorilla beringei*, *Gorilla gorilla*, *Pan paniscus*, *Pan troglodytes*, *Pongo abelii*, *Pongo pygmaeus*, and some modern human samples from different ancestries (Prado-Martinez et al. 2013). We converted the *FADS* region from hg19 to hg18 (11: 61,300,075–61,348,212) through the use of SNP rs IDs. We then phased the data using shapeit (Delaneau et al. 2012) with default settings and constructed a haplotype network as detailed in Recombination Mapping of the Materials and Methods section.

### Tree Analysis

To convert the haplotype network into a tree, we calculated pairwise differences between each haplotype to form a matrix of differences between all haplotypes in the high-coverage data set. Phylip neighbor v 3.68 was then used to form a Neighbor-Joining tree based on the matrix of differences between each haplotype (Felsenstein 2005). To assign confidence that each node is correct, we performed 500 bootstraps by randomly sampling, with replacement, each base over the entire haplotype length for each haplotype. Phylip consense (Felsenstein 2005) was used to calculate a consensus tree from the 500 bootstraps with the human–chimpanzee ancestral reconstructed reference sequence (Altshuler et al. 2015) set as the root of the tree. We then used MEGA7 to plot the consensus tree (Kumar et al. 2016).

### Derived Haplogroup TMRCA Calculations

To determine when the derived haplogroup arose, we calculated the TMRCA based on the degree of differentiation from a human ancestral sequence described in the following equation (Coop et al. 2008):

(2) $$\mathrm{TMRCA}=\left(\frac{M}{A_{\mathrm{HC}}}\right)\;\times 2\;\times T_{\mathrm{HC,}}$$

where M is the average number of differences between all human derived haplotypes and the human–chimpanzee ancestral reconstructed reference sequence (Altshuler et al. 2015) over the entire haplogroup, at only high-quality ancestral reconstructed reference sites (47,820 bases out of the total haplogroup length of 48,408 bases). Only mutations that followed the infinite sites model (Kimura 1969) in modern humans were used for this analysis, meaning only mutations found in the derived haplotypes and not present in the ancestral haplotypes were analyzed. $A_{\mathrm{HC}}$ represents the local human chimpanzee divergence value, which we calculated to be 0.829%, by using liftover (Hinrichs et al. 2006) to determine the hg19 *FADS* haplogroup coordinates in the panTro-4 reference genome downloaded from the UCSC Genome Browser (Chimpanzee Sequencing Analysis Consortium 2005; Tyner et al. 2017). We then aligned the chimpanzee and human reference sequences for the two regions with Clustal Omega 2.1 (Sievers et al. 2014) and calculated the percentage of mutations between the human and chimpanzee. The value $T_{\mathrm{HC}}$ corresponds to the time of the human–chimpanzee divergence, which we specified as 6,500,000 Ma (Hedges et al. 2006, 2015; Kumar and Hedges 2011). We then calculated the variance and SD of the TMRCA for all derived haplotypes, and applied the framework by Hudson (Hudson 2007) to calculate the 95% confidence interval. This analysis only used the high-coverage data set which included modern human invariant positions. We assessed the impact of differing human–chimpanzee divergence values by keeping M and $A_{\mathrm{HC}}$ constant while varying $T_{\mathrm{HC}}$ between 5,000,000 and 7,000,000 Ya.

## Results

### Organization of the *FADS* Gene Region

Recombination mapping showed that there is one major haplotype for both the ancestral and derived haplogroup over the entire *FADS* gene region based on rs174537 (supplementary fig. S1, Supplementary Material online). However, prior research showed that there were two subregions within the *FADS* region, and that the region chr11: 61,567,753–61,606,683 was associated with increased biosynthesis of LC-PUFAs (Ameur et al. 2012). We redefined this region to be chr11: 61,543,499–61,591,907 through PLINK LD block formation, using the Peruvian Genome project samples to isolate the largest LD block. With the high-coverage GeneSTAR and Peruvian Genome Project samples, this region contained 43 SNPs. When intersecting with the 1000 Genomes samples, this region only contained 38 SNPs (supplementary table S1, Supplementary Material online) in the region: chr11: 61,543,499–61,591,636.

### Ancestral and Derived Haplogroup Proportion

Global haplogroup proportions confirmed that African populations were nearly fixed for the derived haplogroup and Eurasia was polymorphic (Mathias et al. 2011, 2012; Ameur et al. 2012) (fig. 1*A*). The GeneSTAR African and European Americans had similar haplogroup proportions as populations in the 1000 Genomes Project from those same regions. Siberian and the Peruvian Genome Project populations had the lowest average derived haplogroup proportions. The 1000 Genomes Native American ancestry populations had a higher ancestral haplogroup frequency than Eurasian populations, but were not fixed for the ancestral haplogroup. Ancestral haplogroup proportions were greater in the mestizo Peruvians than any 1000 Genomes population, and Native American identifying populations had an even greater ancestral haplogroup proportion than the mestizo Peruvians (fig. 1*A*). Although, neither Peruvian population was fixed for the ancestral haplogroup, which leads to the hypothesis that European and African admixture impacted the *FADS* haplogroup frequency in the Americas.

To examine admixture dynamics in populations from the Americas, we calculated local ancestry in all admixed 1000 Genomes African American and mestizo populations in addition to GeneSTAR individuals and Peruvians with <99% Native American ancestry. This showed that the ancestral haplogroup is nearly fixed in Native American ancestry as 97.44% of 386 haplotypes have the ancestral haplogroup (fig. 1*B*). When we restricted our calculations to individuals with unambiguous ancestry (ie homozygous for a single ancestry) we found Africa is 99.74% for the derived and Native American is 99.54% for the ancestral haplogroup (supplementary fig. S2, Supplementary Material online), which further shows that both ancestries are nearly fixed for the derived or ancestral haplogroup. European local ancestry haplotypes’ derived haplogroup proportions were consistant with the observed European population haplogroup proportion patterns (fig. 1 and supplementary fig. S2, Supplementary Material online).

### Selection in Native Americans and Siberian Genomes Analysis

**Fig. 2.**
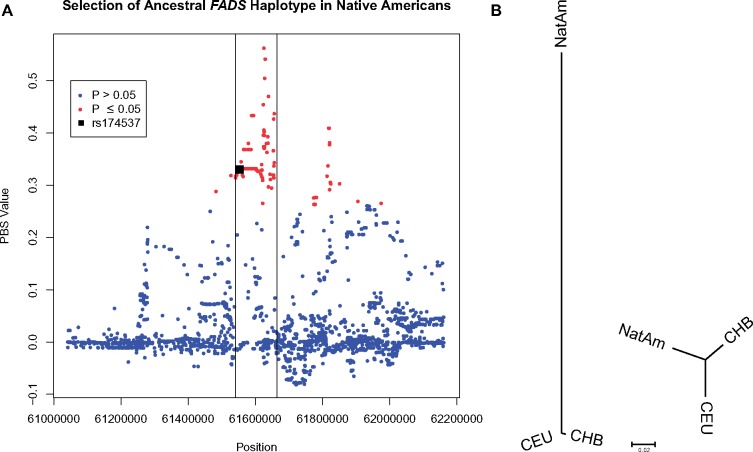
—Signatures of selection in the *FADS* region in Native Americans. (*A*) PBS values from chr11: 61,044,208–62,163,145. The black lines mark the *FADS* gene region and the black square represents the PBS value for rs174537 (*P* = 0.0001242) while red circles represent *P* values that are ≤ 0.05 and blue circles represent *P* values that are >0.05, after correction for multiple hypothesis testing. (*B*) Representation of rs174537 PBS values (left) and the genome wide average PBS values (right). Branch lengths are the PBS values for each population.

We sought to perform a replication analysis of Fumagalli et al. (2015) and Amorim et al. (2017) to determine if the *FADS* region showed signs of positive selection in Peruvians. The rs174537 SNP, and the surrounding *FADS* region, showed evidence of being under positive selection for the ancestral haplogroup in Native Americans relative to the CHB and CEU (PBS = 0.33, *P* = 0.0001242) (fig. 2). In Siberia, we found a sigificant correlation independent of European admixture (β =  0.01016, *R*^2^ = 0.3929, *P* = 5.06×10^−5^) such that the ancestral haplogroup is at a higher proportion in more Northern regions (supplementary figs. S3 and S4, Supplementary Material online), and is a genome wide-outlier (*P* = 0.03).

We also tested if selection is ongoing in Native Americans. Therefore, we simulated the demographic history of an allele with varying selection pressure following a bottleneck at the initial entrance into the Americas at 16,000 Ya (Gravel et al. 2013). Simulating with selection ending at the bottleneck showed that an allele would only go to fixation if the allele frequency at the time of the bottleneck was already close to fixation (90–95%). If the allele frequency was not already close to fixation, simulations with a selection pressure of 0.01 for the entire time since the bottleneck resulted in a high proportion of the allele rising to fixation. Furthermore, the magnitude of the bottlenck also impacted the frequency of fixation as the strongest bottleneck, in combination with selection, resulted in the highest proportion of fixation (supplementary table S2, Supplementary Material online).

### Ancient Humans

To better understand the evolution of this *FADS* haplogroup, we tested if ancient humans followed the pattern of haplogroup proportions seen in modern humans. The ancient African Mota individual (Llorente et al. 2015) was found to be homozygous for the derived haplogroup (fig. 1). Analysis of 19 Neolithic and Bronze age Europeans (Gamba et al. 2014; Lazaridis et al. 2014; Olalde et al. 2014; Haak et al. 2015; Mathieson et al. 2015) revealed the haplogroup to be polymorphic in ancient Europe. Whereas, ancient Siberian (Fu et al. 2014; Raghavan et al. 2014), Eskimo (Rasmussen et al. 2010), and Native American (Rasmussen et al. 2014) genomes were found to be homozygous for the ancestral haplogroup (supplementary table S3, Supplementary Material online).

### *FADS* Haplotype Topography and TMRCA

**Fig. 3.**
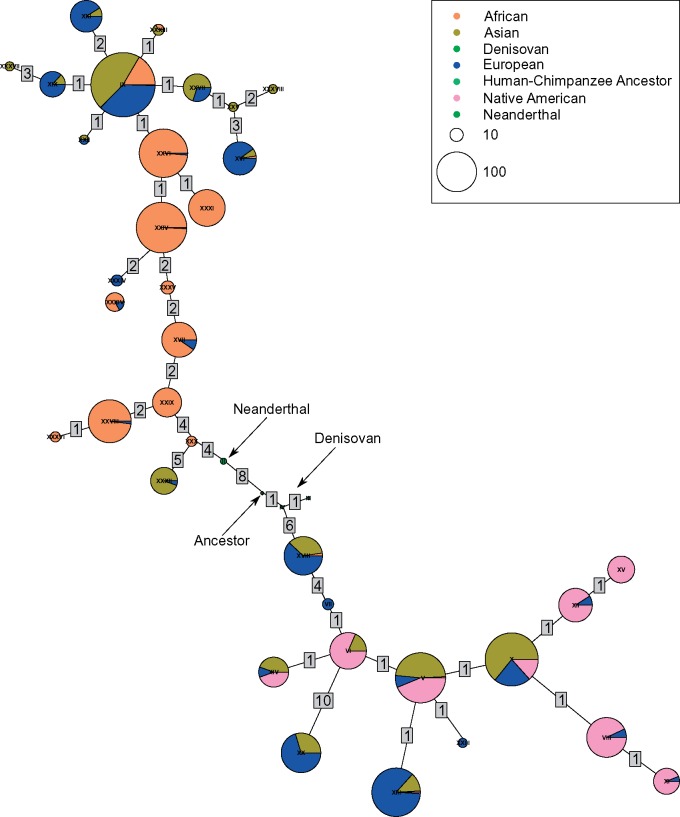
—Haplotype network of chr11: 61,543,499–61,591,636 colored by local ancestry calls. Asian ancestry is assigned to 1000 Genomes populations labeled as being either East or South Asian by the 1000 Genomes Project. African, European, and Native American ancestry is assigned to the admixed populations from their local ancestry estimates, however source and nonadmixed populations are assigned their global population label determined by the 1000 Genomes Project or the Peruvian Genome Project. Numbers present on each link represent the number of mutations that separate the two haplotypes.

**Fig. 4.**
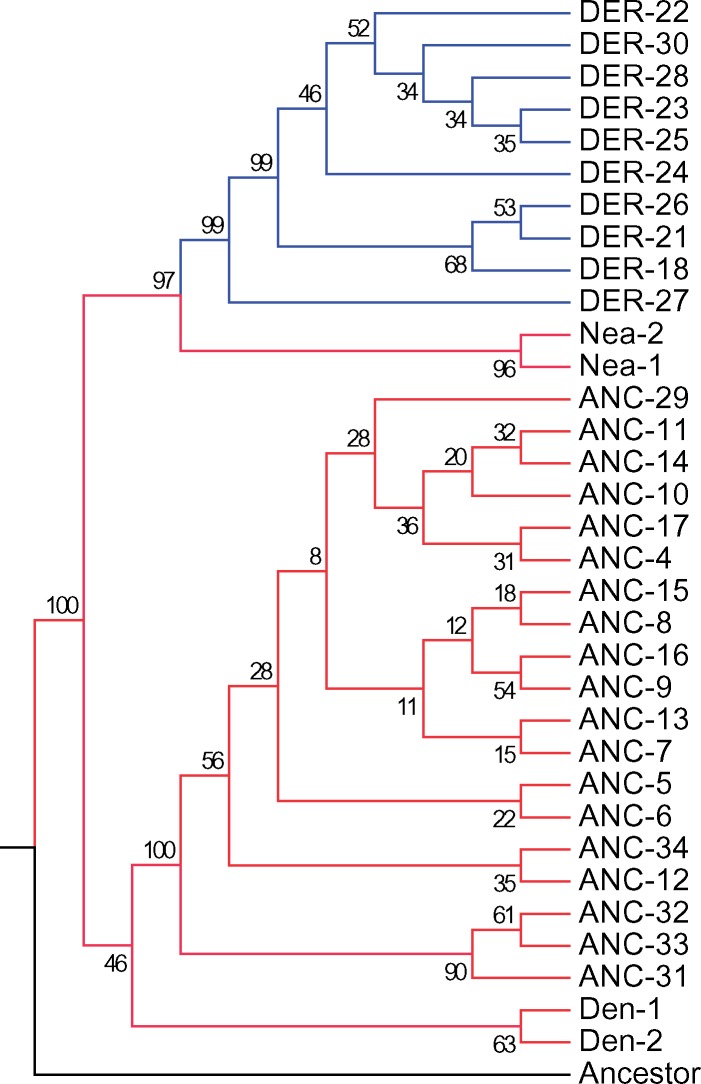
—Tree representation of major haplotype networks, including invariant sites. The numbers on each node represent the percent that node was formed out of 500 bootstraps. Haplotype number is abbreviated as Hap# with the number coming from the Roman numeral in the high-coverage haplotype network (supplementary fig. S7, Supplementary Material online). Nea-1 = Haplotype 2, Nea-2 = Haplotype 3, Den-1 = Haplotype 19, Den-2 = Haplotype 20, and Ancestor = Haplotype 1 (supplementary fig. S7, Supplementary Material online).

Haplotype networks of modern humans and archaic hominins revealed two main clusters, a derived and ancestral cluster, where the derived haplogroup has a TMRCA of 688,474 Ya (95% confidence interval = 635,978–743,052) and is robust to different values of human–chimpanzee divergence estimates (supplementary fig. S8, Supplementary Material online). Interestingly, there were two modern human ancestral haplotypes (HAP# XXX, XXXII) that were closer to the derived haplogroup than to the ancestral haplogroup (supplementary fig. S5, Supplementary Material online). Haplotype XXXII contains individuals with Asian or European ancestry (fig. 3). Haplotype XXX is entirely of African ancestry and primarily has individuals from continental African populations. However, there is one Colombian individual whose other haplotype is of European ancestry (fig. 3 and supplementary fig. S5, Supplementary Material online). The majority of Native American haplotypes appeared in the ancestral cluster and the majority of African haplotypes were in the derived cluster, with Eurasian haplotypes distributed among both haplogroups (fig. 3 and supplementary fig. S5, Supplementary Material online). There was not one unique signature representing all Native American ancestral haplotypes, Eurasian, or African derived haplotypes (table 1). A core haplotype for these three can be formed, although the core haplotypes contain variants found in other ancestral or derived haplotypes (table 1). In addition, the archaic hominins are intermediate between the two haplogroups, although each hominin’s haplotypes are more closely related to one of the haplogroups than to the other hominin (fig. 3 and supplementary fig. S5, Supplementary Material online). The Neanderthal haplotypes are more closely related to the modern human derived haplogroup than to the modern human ancestral haplogroup, and the Denisovan haplotypes cluster closer to the modern human ancestral haplogroup, although with poor bootstrap support (figs. 3 and 4). When forming a haplotype network with human and nonhuman great apes, we saw that there is greater genetic variation in the *FADS* region among nonhuman great apes. In addition, we found that all nonhuman great apes are fixed for the ancestral allele at rs174537 and are greatly different from all modern human samples (supplementary fig. S6, Supplementary Material online).

**Table 1:** Core Haplotypes

Category	Sequence
Ancestral	CCnnnAnCC **T** CCnnnnnnnnnnnnnnnnnnnnnnnnnn
Derived	nnnGnnnnn **G** nTGnnCCCTnGTCTAATGCAAGnTAnnC
Native American Ancestral	CCGnTAGCC **T** CCATCAGTCGACnCGGCnTGnACCGGGn
African Derived	nnnGnnnnA **G** nTGGTCCCTnGTCTAATGCAAGnTAnnC
Asian Derived	nnnGnnnnn **G** TTGnnCCCTCGTCTAATGCAAGTTAAAC
Asian Ancestral	CCGGnAnCC **T** CCnnnnnnnnnnnnnnnGnnnnnnnnnn
European Derived	nnnGnGnnA **G** nTGnnCCCTnGTCTAATGCAAGnTAnnC
European Ancestral	CCGnnAnCC **T** CCnnnnnnnnnnnnnnnnnnnnnnnnnn

Note.—Capital bases are found in all haplotypes for each category. Lower case “n” represents a variable base in each haplogroup category. The bold base represents rs174537. The base number (1–38, left to right) corresponds to the variants found in supplementary table S1, Supplementary Material online.

## Discussion

The differential clustering of the Neanderthal and Denisovan haplotypes with modern humans does not support archaic introgression of the ancestral haplogroup. Instead, the great differentiation between the ancestral and derived haplogroups and the ancient TMRCA supports the alternative hypothesis that the *FADS* gene region is old in the hominin lineage and that the derived haplogroup began to form around the divergence between the three hominins (table 1, figs. 3 and 4, and supplementary fig. S5, Supplementary Material online). The TMRCA of the derived haplotypes is within the modern human-archaic hominin divergence period (555,000–765,000 Ya) (Prufer et al. 2014), which is consistent with the antiquity of this haplotype, except when a human–chimpanzee divergence time of ≤5,192,642 Ya was used (supplementary fig. S8, Supplementary Material online).

The haplotype topography and tree (fig. 4 and supplementary fig. S5, Supplementary Material online) indicated that the TMRCA should predate the divergence of these hominins. Since both the ancestral and derived haplotypes were under positive selection (fig. 2) (Mathias et al. 2012; Fumagalli et al. 2015; Amorim et al. 2017; Buckley et al. 2017), we likely underestimated the TMRCA of the derived haplotypes, because positive selection causes a reduction in the diversity of variants within a haplotype (Sabeti et al. 2006). As a result, it is possible that the actual TMRCA of the derived haplotypes predates the modern-archaic human divergence and supports our hypothesis that the derived haplogroup formed during or prior to the divergence of these three hominins.

The TMRCA we present is older than previous estimates (Ameur et al. 2012; Mathias et al. 2012). The differences in TMRCA calculations are likely due to each study using a slightly different genomic segment of the *FADS* region (Ameur et al. 2012; Mathias et al. 2012). In addition, we used a greater number of samples to calculate the TMRCA than Ameur et al. (2012), and this likely increased haplotype diversity and led to an older TMRCA. Furthermore, we only used high-coverage sequences (Mathias et al. 2011; Harris et al. 2018) since this allowed us to identify more variants and therefore lead to a greater estimate of TMRCA than prior studies (Ameur et al. 2012; Mathias et al. 2012).

While prior work was done on a few Native American ancestry samples (Mathias et al. 2011, 2012; Ameur et al. 2012), with 386 Native American haplotypes, we determined that the Native American populations are nearly fixed for the ancestral haplogroup and replicated a signal of positive selection (Amorim et al. 2017) (figs. 1*B* and 2 and supplementary fig. S2, Supplementary Material online). This is puzzling due to the potentially detrimental health effects that could arise from having a reduced capability to synthesize LC-PUFAs that are vital to brain and immune system development (Marszalek and Lodish 2005; Calder 2013). However, Fumagalli et al. (2015) suggested that the ancestral haplogroup could be linked to cold weather adaptation possibly as a response to dietary restrictions of a cold climate, although the exact selection pressure is unknown. We found that Siberian populations have a high proportion for the ancestral haplogroup and that the proportion increases the further north a population lives in Asia, which is consistent with this hypothesis (supplementary fig. S4, Supplementary Material online). According to one recent hypothesis, Native American ancestors remained isolated (possibly in Beringia) for up to 10,000 years prior to migrating into North America (Tamm et al. 2007; Gravel et al. 2013; Raghavan et al. 2015; Moreno-Mayar, Potter et al. 2018; Moreno-Mayar, Vinner et al. 2018), where there would have been a strong selection pressure for genetic variants that assisted in adapting to dietary demands from a cold weather climate. There have also been other variants identified to help populations adapt to the cold climate that involve metabolic processes in the mitochondria. While these variants identified are derived as opposed to ancestral, it provides important evidence that genetic variants do assist with cold weather adaptation (Mishmar et al. 2003).

In addition, some have posited that Native American ancestors spread into North America through a coastal route (Pedersen et al. 2016). This also could have facilitated reduced selection for LC-PUFA biosynthesis due to the fact that large quantities of LC-PUFAs would have been found in seafood along the coast (Horrocks and Yeo 1999). Eventually Native American populations left the coast and moved inland. The persistence of the ancestral haplogroup could then be explained by Native American populations’ low-effective population sizes (Harris et al. 2018). Their low-effective population size would require an extremely high selection pressure to reduce the haplogroup frequency in a population already with the haplogroup at near fixation (Hartl and Clark 2007). However, the actual selection pressure for the ancestral haplogroup in Native Americans has not been confirmed. It is possible that the cold climate and a diet high in LC-PUFAs is not the section pressure, and therefore requires future studies that include phenotype data from Native American ancestry populations, as well as functional analyses in model organisms.

An alternative hypothesis is that the signal of positive selection we detected in modern Native Americans is a remnant of old positive selection for the ancestral haplogroup that occurred shortly after the Out-of-Africa expansion, as suggested by Mathieson and Mathieson (2018). In this scenario, selection would not be recent and possibly not linked to cold weather adaptation. Simulation analysis suggested that, if the allele frequency was not pushed to at least 90% from selection in either the Bering Strait or shortly after migrating out of Africa, then continuing selection of the ancestral haplogroup would be required to observe the near fixation in modern Native American populations (supplementary table S2, Supplementary Material online). Therefore, it is essential that we understand the haplogroup frequency in the Native American founding population, which can be accomplished through ancient DNA analysis of Siberian and East Asian populations. We found that the few ancient genomes from Siberia and the Americas, which included samples that are representative of the first migration into the Americas (Raghavan et al. 2014; Rasmussen et al. 2014), are homozygous for the ancestral haplogroup (supplementary table S3, Supplementary Material online). Therefore, ancient DNA indicates that the ancestral haplogroup frequency in the Native American founding population was extremely high and is consistent with ancient positive selection. Although our sample size of ancient humans from Siberia and the Americas is small (*n* = 4), a recent study of a much larger ancient Native American DNA data set (*n* = 49) found similar results (Posth et al. 2018). We still do not have a clear picture of the ancient allele frequency, and as a result, we cannot differentiate between a scenario of ancient positive selection shortly after the Out-of-Africa expansion or positive selection continuing into the founding of the Americas. Future efforts should aim to develop large ancient genome data sets from Asia and the Americas, such as in Europe (Mathieson et al. 2015), to better understand the Native American founding population’s *FADS* genetic architecture.

One complication regarding the *FADS* region is that the causal variant for altering the efficiency of LC-PUFA biosynthesis has not yet been identified. Strong associations are known between an individual’s genotype at rs174537 and the efficiency of 18 C to LC-PUFAs conversion (Mathias et al. 2011; Sergeant et al. 2012). However, likely due to extremely high LD in this region (supplementary fig. S1, Supplementary Material online), the causal variant cannot be determined. We found one additional variant to rs174537, rs102274 (chr11: 61557826), that was fixed in opposite directions for the ancestral and derived haplogroups (table 1). Therefore, this is a potential causal variant in the *FADS* cluster that deserves further analysis to determine if there is any functional importance in the *FADS* region. We also identify less likely candidates that are fixed in either the ancestral or derived haplogroups, while being polymorphic in the opposite haplogroup (table 1, represented by capital letters in one haplogroup and an “n” in the other).

LC-PUFAs are essential for a wide range of human biological functions (Mead et al. 1953; Steinberg et al. 1956; Alessandri et al. 2004; Kitajka et al. 2004). A reduced capacity to synthesize LC-PUFAs has the potential to be a public health risk for modern populations with high Native American ancestry. For example, the *n*-3 LC-PUFA, docosahexaenoic acid is known to be critical for brain function throughout the human life span, but its accumulation is especially important to healthy brain development during gestation and infancy (Kitajka et al. 2004; Marszalek and Lodish 2005). In the brain, docosahexaenoic acid has a wide range of neurological functions including membrane integrity, neurotransmission, neurogenesis and synaptic plasticity, membrane receptor function and signal transduction (Marszalek and Lodish 2005). Additionally, *n*-3 LC-PUFAs such as docosahexaenoic acid, docosapentaenoic acid and eicosapentaenoic acid and their metabolites have potent anti-inflammatory properties (Marszalek and Lodish 2005; Calder 2013). There has been a dramatic increase in dietary exposure to linoleic acid (an *n*-6 18C-PUFA) due to the addition of vegetable oil products to the modern Western diet over the past 50 years (Chilton et al. 2014). This increase has shifted the ratio of *n*-6 to *n*-3 18C-PUFAs ingested to greater than 10:1 which assures that *n*-6 linoleic acid and not *n*-3 α-linolenic acid is the primary substrate that enters the LC-PUFA biosynthetic pathway thereby producing arachidonic acid and not eicosapentaenoic acid, docosapentaenoic acid, and docosahexaenoic acid (Chilton et al. 2014).

Therefore, the critical question from a gene–diet interaction perspective is; does the near fixation of the ancestral haplogroup with its limited capacity to synthesize LC-PUFAs in Native American ancestry individuals together with an overwhelming exposure of linoleic acid relative to α-linolenic acid entering the biosynthetic pathway give rise to *n*-3 LC-PUFA deficiencies and resulting diseases/disorders in Native American ancestry populations? Simply stated, what are the sources of *n*-3 LC-PUFAs for Native American ancestry individuals during critical periods of brain development and as anti-inflammatory mediators (Simopoulos 1999)? Questions such as these indicate that future research is needed to assess circulating and tissue total PUFA levels in Native American ancestry individuals. If these individuals are found to have low LC-PUFA levels, then they will be an important cohort to study the risk of LC-PUFA deficiencies and related dietary interventions in this area could provide a substantive benefit to Native American ancestry populations’ medical care.

## Supplementary Material

Supplementary data are available at *Genome Biology and Evolution* online.

## Supplementary Material

Supplementary_Material_evz071

## References

[evz071-B1] Alessandri J-M, et al 2004 Polyunsaturated fatty acids in the central nervous system: evolution of concepts and nutritional implications throughout life. Reprod Nutr Dev. 44(6):509–538. 15762297 10.1051/rnd:2004063

[evz071-B2] Alexander DH, Novembre J, Lange K. 2009 Fast model-based estimation of ancestry in unrelated individuals. Genome Res. 19(9):1655–1664. 19648217 10.1101/gr.094052.109 PMC2752134

[evz071-B3] Altshuler DM, et al 2015 A global reference for human genetic variation. Nature 526:68. 26432245 10.1038/nature15393 PMC4750478

[evz071-B4] Ameur A, et al 2012 Genetic adaptation of fatty-acid metabolism: a human-specific haplotype increasing the biosynthesis of long-chain omega-3 and omega-6 fatty acids. Am J Hum Genet. 90(5):809–820. 22503634 10.1016/j.ajhg.2012.03.014 PMC3376635

[evz071-B5] Amorim CE, et al 2017 Genetic signature of natural selection in first Americans. Proc Natl Acad Sci U S A. 114(9):2195–2199. 28193867 10.1073/pnas.1620541114 PMC5338486

[evz071-B6] Berkeley Earth. Land + ocean data, annual average temperature with air temperatures at sea ice. Available from: http://berkeleyearth.org/land-and-ocean-data/

[evz071-B7] Broad Institute. 2014. Picard. Available from: http://broadinstitute.github.io/picard/; last accessed February 24, 2019

[evz071-B8] Buckley MT, et al 2017 Selection in Europeans on fatty acid desaturases associated with dietary changes. Mol Biol Evol. 34(6):1307–1318. 28333262 10.1093/molbev/msx103 PMC5435082

[evz071-B9] Calder PC. 2013 Long chain fatty acids and gene expression in inflammation and immunity. Curr Opin Clin Nutr Metab Care. 16(4):425–433. 23657154 10.1097/MCO.0b013e3283620616

[evz071-B10] Chang CC, et al 2015 Second-generation PLINK: rising to the challenge of larger and richer datasets. Gigascience 4:7. 25722852 10.1186/s13742-015-0047-8 PMC4342193

[evz071-B11] Chilton F, et al 2014 Diet-gene interactions and PUFA metabolism: a potential contributor to health disparities and human diseases. Nutrients 6(5):1993–2022. 24853887 10.3390/nu6051993 PMC4042578

[evz071-B12] Chimpanzee Sequencing Analysis Consortium. 2005 Initial sequence of the chimpanzee genome and comparison with the human genome. Nature 437:69–87. 16136131 10.1038/nature04072

[evz071-B13] Coop G, Bullaughey K, Luca F, Przeworski M. 2008 The timing of selection at the human FOXP2 gene. Mol Biol Evol. 25(7):1257–1259. 18413354 10.1093/molbev/msn091 PMC2429961

[evz071-B14] Danecek P, et al 2011 The variant call format and VCFtools. Bioinformatics 27(15):2156–2158. 21653522 10.1093/bioinformatics/btr330 PMC3137218

[evz071-B15] Delaneau O, Marchini J, Zagury JF. 2012 A linear complexity phasing method for thousands of genomes. Nat Methods. 9(2):179–181. 10.1038/nmeth.1785 22138821

[evz071-B16] Felsenstein J. 2005 PHYLIP (Phylogeny Inference Package) version 3.6. Seattle: Department of Genome Sciences, University of Washington.

[evz071-B17] Fu Q, et al 2014 Genome sequence of a 45,000-year-old modern human from western Siberia. Nature 514(7523):445–449. 25341783 10.1038/nature13810 PMC4753769

[evz071-B18] Fumagalli M, et al 2015 Greenlandic Inuit show genetic signatures of diet and climate adaptation. Science 349(6254):1343–1347. 26383953 10.1126/science.aab2319

[evz071-B19] Gamba C, et al 2014 Genome flux and stasis in a five millennium transect of European prehistory. Nat Commun. 5:5257. 25334030 10.1038/ncomms6257 PMC4218962

[evz071-B20] Gautier M, Vitalis R. 2012 rehh: an R package to detect footprints of selection in genome-wide SNP data from haplotype structure. Bioinformatics 28(8):1176–1177. 22402612 10.1093/bioinformatics/bts115

[evz071-B21] Gravel S, et al 2013 Reconstructing native American migrations from whole-genome and whole-exome data. PLoS Genet. 9(12):e1004023. 24385924 10.1371/journal.pgen.1004023 PMC3873240

[evz071-B22] Haak W, et al 2015 Massive migration from the steppe was a source for Indo-European languages in Europe. Nature 522(7555):207–211. 25731166 10.1038/nature14317 PMC5048219

[evz071-B23] Harris DN, et al 2018 Evolutionary genomic dynamics of Peruvians before, during, and after the Inca Empire. Proc Natl Acad Sci U S A. 115(28):E6526–E6535. 29946025 10.1073/pnas.1720798115 PMC6048481

[evz071-B24] Hartl DL, Clark AG. 2007 Principles of population genetics. Sunderland, MA: Sinauer associates.

[evz071-B25] Hedges SB, Dudley J, Kumar S. 2006 TimeTree: a public knowledge-base of divergence times among organisms. Bioinformatics 22(23):2971–2972. 17021158 10.1093/bioinformatics/btl505

[evz071-B26] Hedges SB, Marin J, Suleski M, Paymer M, Kumar S. 2015 Tree of life reveals clock-like speciation and diversification. Mol Biol Evol. 32(4):835–845. 25739733 10.1093/molbev/msv037 PMC4379413

[evz071-B27] Hinrichs AS, et al 2006 The UCSC Genome Browser Database: update 2006. Nucleic Acids Res. 34(90001):D590–D598. 16381938 10.1093/nar/gkj144 PMC1347506

[evz071-B28] Horrocks LA, Yeo YK. 1999 Health benefits of docosahexaenoic acid (DHA). Pharmacol Res. 40(3):211–225. 10479465 10.1006/phrs.1999.0495

[evz071-B29] Howe P, Meyer B, Record S, Baghurst K. 2006 Dietary intake of long-chain omega-3 polyunsaturated fatty acids: contribution of meat sources. Nutrition 22(1):47–53. 16289978 10.1016/j.nut.2005.05.009

[evz071-B30] Hudson RR. 2007 The variance of coalescent time estimates from DNA sequences. J Mol Evol. 64(6):702–705. 17487522 10.1007/s00239-006-0261-1

[evz071-B31] Huerta-Sanchez E, et al 2014 Altitude adaptation in Tibetans caused by introgression of Denisovan-like DNA. Nature 512:194–197. 25043035 10.1038/nature13408 PMC4134395

[evz071-B301] Jones PD, New M, Parker DE, Martin S, Rigor IG. 1999. Surface air temperature and its changes over the past 150 years. Reviews of Geophysics 37:173–199.

[evz071-B32] Kimura M. 1969 The number of heterozygous nucleotide sites maintained in a finite population due to steady flux of mutations. Genetics 61:893–903. 5364968 10.1093/genetics/61.4.893 PMC1212250

[evz071-B33] Kitajka K, et al 2004 Effects of dietary omega-3 polyunsaturated fatty acids on brain gene expression. Proc Natl Acad Sci U S A. 101(30):10931–10936. 15263092 10.1073/pnas.0402342101 PMC503722

[evz071-B34] Kumar S, Hedges SB. 2011 TimeTree2: species divergence times on the iPhone. Bioinformatics 27(14):2023–2024. 21622662 10.1093/bioinformatics/btr315 PMC3129528

[evz071-B35] Kumar S, Stecher G, Tamura K. 2016 MEGA7: Molecular Evolutionary Genetics Analysis version 7.0 for bigger datasets. Mol Biol Evol. 33(7):1870–1874. 27004904 10.1093/molbev/msw054 PMC8210823

[evz071-B36] Lazaridis I, et al 2014 Ancient human genomes suggest three ancestral populations for present-day Europeans. Nature 513(7518):409–413. 25230663 10.1038/nature13673 PMC4170574

[evz071-B37] Leinonen R, Sugawara H, Shumway M, International Nucleotide SDC. 2011 The sequence read archive. Nucleic Acids Res. 39(Database):D19–D21. 21062823 10.1093/nar/gkq1019 PMC3013647

[evz071-B38] Li H. 2013. Aligning sequence reads, clone sequences and assembly contigs with BWA-MEM. arXiv 1303.3997v2.

[evz071-B39] Li H, et al 2009 The Sequence Alignment/Map format and SAMtools. Bioinformatics 25(16):2078–2079. 19505943 10.1093/bioinformatics/btp352 PMC2723002

[evz071-B40] Li JZ, et al 2008 Worldwide human relationships inferred from genome-wide patterns of variation. Science 319(5866):1100–1104. 18292342 10.1126/science.1153717

[evz071-B41] Liu S, et al 2018 Genomic analyses from non-invasive prenatal testing reveal genetic associations, patterns of viral infections, and Chinese population history. Cell 175(2):347–359. e314. 10.1016/j.cell.2018.08.016 30290141

[evz071-B42] Llorente MG, et al 2015 Ancient Ethiopian genome reveals extensive Eurasian admixture throughout the African continent. Science 350(6262):820–822. 26449472 10.1126/science.aad2879

[evz071-B43] Manichaikul A, et al 2010 Robust relationship inference in genome-wide association studies. Bioinformatics 26(22):2867–2873. 20926424 10.1093/bioinformatics/btq559 PMC3025716

[evz071-B44] Maples BK, Gravel S, Kenny EE, Bustamante CD. 2013 RFMix: a discriminative modeling approach for rapid and robust local-ancestry inference. Am J Hum Genet. 93(2):278–288. 23910464 10.1016/j.ajhg.2013.06.020 PMC3738819

[evz071-B45] Marszalek JR, Lodish HF. 2005 Docosahexaenoic acid, fatty acid-interacting proteins, and neuronal function: breastmilk and fish are good for you. Annu Rev Cell Dev Biol. 21(1):633–657. 16212510 10.1146/annurev.cellbio.21.122303.120624

[evz071-B46] Mathias RA, et al 2012 Adaptive evolution of the FADS gene cluster within Africa. PLoS One 7(9):e44926. 23028684 10.1371/journal.pone.0044926 PMC3446990

[evz071-B47] Mathias RA, Pani V, Chilton FH. 2014 Genetic variants in the FADS gene: implications for dietary recommendations for fatty acid intake. Curr Nutr Rep. 3(2):139–148. 24977108 10.1007/s13668-014-0079-1 PMC4070521

[evz071-B48] Mathias RA, et al 2011 The impact of FADS genetic variants on omega6 polyunsaturated fatty acid metabolism in African Americans. BMC Genet. 12(1):50. 21599946 10.1186/1471-2156-12-50 PMC3118962

[evz071-B49] Mathieson I. 2015 FADS genes, selection and diet. Available from: http://mathii.github.io/research/2015/12/14/fads1-selection-and-diet; last accessed February 24, 2019.

[evz071-B50] Mathieson I, et al 2015 Genome-wide patterns of selection in 230 ancient Eurasians. Nature 528(7583):499–503. 26595274 10.1038/nature16152 PMC4918750

[evz071-B51] Mathieson S, Mathieson I. 2018 FADS1 and the timing of human adaptation to agriculture. Mol Biol Evol. 35(12):2957–2970. 30272210 10.1093/molbev/msy180 PMC6278866

[evz071-B52] McKenna A, et al 2010 The Genome Analysis Toolkit: a MapReduce framework for analyzing next-generation DNA sequencing data. *Genome* Res. 20:1297–1303. 10.1101/gr.107524.110 PMC2928508 20644199

[evz071-B53] Mead JF, Steinberg G, Howton DR. 1953 Metabolism of essential fatty acids; incorporation of acetate into arachidonic acid. J Biol Chem. 205(2):683–689. 13129246

[evz071-B54] Mendez FL, Watkins JC, Hammer MF. 2012 A haplotype at STAT2 Introgressed from Neanderthals and serves as a candidate of positive selection in Papua New Guinea. Am J Hum Genet. 91(2):265–274. 22883142 10.1016/j.ajhg.2012.06.015 PMC3415544

[evz071-B55] Meyer M, et al 2012 A high-coverage genome sequence from an archaic Denisovan individual. Science 338(6104):222–226. 22936568 10.1126/science.1224344 PMC3617501

[evz071-B56] Mishmar D, et al 2003 Natural selection shaped regional mtDNA variation in humans. Proc Natl Acad Sci U S A. 100(1):171–176. 12509511 10.1073/pnas.0136972100 PMC140917

[evz071-B57] Moreno-Mayar JV, Potter BA, et al 2018 Terminal Pleistocene Alaskan genome reveals first founding population of Native Americans. Nature 553(7687):203–207. 29323294 10.1038/nature25173

[evz071-B58] Moreno-Mayar JV, Vinner L, et al 2018 Early human dispersals within the Americas. *Science* 362:eaav2621. 10.1126/science.aav2621 30409807

[evz071-B59] Nakamura MT, Nara TY. 2004 Structure, function, and dietary regulation of delta6, delta5, and delta9 desaturases. Annu Rev Nutr. 24(1):345–376. 15189125 10.1146/annurev.nutr.24.121803.063211

[evz071-B60] Olalde I, et al 2014 Derived immune and ancestral pigmentation alleles in a 7,000-year-old Mesolithic European. Nature 507(7491):225–228. 24463515 10.1038/nature12960 PMC4269527

[evz071-B61] Paradis E. 2010 pegas: an R package for population genetics with an integrated-modular approach. Bioinformatics 26(3):419–420. 20080509 10.1093/bioinformatics/btp696

[evz071-B62] Paradis E, Claude J, Strimmer K. 2004 APE: Analyses of Phylogenetics and Evolution in R language. Bioinformatics 20(2):289–290. 14734327 10.1093/bioinformatics/btg412

[evz071-B63] Pawlosky RJ, Hibbeln JR, Novotny JA, Salem N Jr. 2001 Physiological compartmental analysis of alpha-linolenic acid metabolism in adult humans. J Lipid Res. 42:1257–1265. 11483627

[evz071-B64] Pedersen MW, et al 2016 Postglacial viability and colonization in North America’s ice-free corridor. Nature 537(7618):45–49. 27509852 10.1038/nature19085

[evz071-B65] Posth C, et al 2018 Reconstructing the deep population history of Central and South America. Cell 175(5):1185–1197 e1122. 30415837 10.1016/j.cell.2018.10.027 PMC6327247

[evz071-B66] Prado-Martinez J, et al 2013 Great ape genetic diversity and population history. Nature 499(7459):471–475. 23823723 10.1038/nature12228 PMC3822165

[evz071-B67] Prufer K, et al 2014 The complete genome sequence of a Neanderthal from the Altai Mountains. Nature 505:43–49. 24352235 10.1038/nature12886 PMC4031459

[evz071-B68] Purcell S, Chang C. PLINK version 1.9. Available from: https://www.cog-genomics.org/plink2; last accessed February 24, 2019.

[evz071-B69] Quinlan AR, Hall IM. 2010 BEDTools: a flexible suite of utilities for comparing genomic features. Bioinformatics 26(6):841–842. 20110278 10.1093/bioinformatics/btq033 PMC2832824

[evz071-B70] Raghavan M, et al 2015 Population genetics. Genomic evidence for the Pleistocene and recent population history of Native Americans. *Science* 349:aab3884. 10.1126/science.aab3884 PMC4733658 26198033

[evz071-B71] Raghavan M, et al 2014 Upper Palaeolithic Siberian genome reveals dual ancestry of Native Americans. Nature 505(7481):87–91. 24256729 10.1038/nature12736 PMC4105016

[evz071-B72] Rasmussen M, et al 2014 The genome of a late Pleistocene human from a Clovis burial site in western Montana. Nature 506(7487):225–229. 24522598 10.1038/nature13025 PMC4878442

[evz071-B73] Rasmussen M, et al 2010 Ancient human genome sequence of an extinct Palaeo-Eskimo. Nature 463(7282):757–762. 20148029 10.1038/nature08835 PMC3951495

[evz071-B74] Sabeti PC, et al 2006 Positive natural selection in the human lineage. Science 312(5780):1614–1620. 16778047 10.1126/science.1124309

[evz071-B75] Sankararaman S, et al 2014 The genomic landscape of Neanderthal ancestry in present-day humans. Nature 507(7492):354–357. 24476815 10.1038/nature12961 PMC4072735

[evz071-B76] Sankararaman S, Mallick S, Patterson N, Reich D. 2016 The combined landscape of Denisovan and Neanderthal ancestry in present-day humans. Curr Biol. 26(9):1241–1247. 27032491 10.1016/j.cub.2016.03.037 PMC4864120

[evz071-B77] Sergeant S, et al 2012 Differences in arachidonic acid levels and fatty acid desaturase (FADS) gene variants in African Americans and European Americans with diabetes or the metabolic syndrome. Br J Nutr. 107(04):547–555. 21733300 10.1017/S0007114511003230 PMC3494092

[evz071-B78] Sievers F, et al 2014 Fast, scalable generation of high-quality protein multiple sequence alignments using Clustal Omega. Mol Syst Biol. 7(1):539. 10.1038/msb.2011.75 PMC3261699 21988835

[evz071-B79] Simonti CN, et al 2016 The phenotypic legacy of admixture between modern humans and Neanderthals. Science 351(6274):737–741. 26912863 10.1126/science.aad2149 PMC4849557

[evz071-B80] Simopoulos AP. 1999 Essential fatty acids in health and chronic disease. Am J Clin Nutr. 70(3 Suppl):560S–569S. 10479232 10.1093/ajcn/70.3.560s

[evz071-B81] Steinberg G, Slaton WH Jr, Howton DR, Mead JF. 1956 Metabolism of essential fatty acids. IV. Incorporation of linoleate into arachidonic acid. J Biol Chem. 220(1):257–264. 13319343

[evz071-B82] Tamm E, et al 2007 Beringian standstill and spread of Native American founders. PLoS One 2(9):e829. 17786201 10.1371/journal.pone.0000829 PMC1952074

[evz071-B83] Tucci S, et al 2018 Evolutionary history and adaptation of a human pygmy population of Flores Island, Indonesia. Science 361(6401):511–516. 30072539 10.1126/science.aar8486 PMC6709593

[evz071-B84] Tyner C, et al 2017 The UCSC Genome Browser database: 2017 update. Nucleic Acids Res. 45(D1):D626–D634. 27899642 10.1093/nar/gkw1134 PMC5210591

[evz071-B85] Vernot B, Akey JM. 2014 Resurrecting surviving Neanderthal lineages from modern human genomes. Science 343(6174):1017–1021. 24476670 10.1126/science.1245938

[evz071-B86] Vernot B, et al 2016 Excavating Neanderthal and Denisovan DNA from the genomes of Melanesian individuals. Science 352(6282):235–239. 26989198 10.1126/science.aad9416 PMC6743480

[evz071-B87] Yi X, et al 2010 Sequencing of 50 human exomes reveals adaptation to high altitude. Science 329:75–78. 20595611 10.1126/science.1190371 PMC3711608

