# Supplementary material for: Evolution of Hominin Polyunsaturated Fatty Acid Metabolism: From Africa to the New World
Source: Genome Biol Evol. 2019 Apr 3;11(5):1417–30. doi: 10.1093/gbe/evz071 (PMC6514828; doi:10.1093/gbe/evz071)
Supplement: Supplementary_Material_evz071 [file supplementary_material_evz071.zip › Supplementary Tables and Figure Legends.docx]

**Supplementary Tables and Figures**

**Tables**

**Additional File 2:** Additional File 2.pdf

**Table S1.** *FADS* Haplogroup SNPs in the low coverage dataset.

**Additional File 6:** Additional File 6.xlsx

**Table S2.** Allele Frequency Simulation

**Additional File 7:** Additional File 7.pdf

**Table S3.** Ancestral and Derived haplogroup proportions in ancient humans.

**Figures**

**Additional File 1:** Additional File 1.pdf

**Figure S1.** Recombination mapping in the *FADS* region. A) Represents haplotype decay of all ancestral haplotypes (n = 1834) and B) represents the derived haplotypes (n = 3860). Ancestral vs derived is determined by SNP rs174537.

**Additional File 3:** Additional File 3.pdf

**Figure S2.** Ancestral and derived haplogroup proportion of individuals with homozygous local ancestry haplotypes (African = 380, European = 450, and Native American ancestry = 216 estimated haplotypes).

**Additional File 4:** Additional File 4.pdf

**Figure S3.** Admixture proportions of Siberian, East and South Asian populations. Admixture analysis with 6 ancestral clusters (K=6) of Siberian, East and South Asian, African (Yoruba) and European (French) populations.

**Additional File 5:** Additional File 5.pdf

**Figure S4.** Ancestral haplogroup proportion, in Siberian, East (EAsia) and South (SAsia) Asian populations in relation to their latitude coordinates. The black line represents the line of best fit from the linear regression lm(proportion ~ European_admixture + latitude).

**Additional File 8:** Additional File 8.pdf

**Figure S5.** Haplotype network of chr11:61,543,499-61,591,636. All haplotypes are at least a count of 4 except for the Neanderthal, Denisovan, and Human-Chimpanzee Ancestor haplotypes. Numbers present on each link represent the number of mutations that separate the two haplotypes.

**Additional File 9:** Additional File 9.pdf

**Figure S6.** Haplotype network of Great Apes over chr11:61,300,075-61,348,212 (hg18). The number of mutations separating each haplotype are designated by the number in each box. All non-human Great Apes are in italics and different human populations are in normal font. Singleton haplotypes are included in this network.

**Additional File 10**: Additional File 10.pdf

**Figure S7.** Haplotype network including all invariant sites in modern humans. All haplotypes have haplotype count > 3 except for the human-chimpanzee ancestor, Neanderthal, and Denisovan haplotypes. Numbers present on each link represent the number of mutations that separate the two haplotypes.

**Additional File 11:** Additional File 11.pdf

**Figure S8.** Derived haplotype TMRCA with Human-Chimpanzee divergence values between 5,000,000 and 7,000,000 ya. The red line indicates the modern-archaic hominin divergence range.
