# Supplementary material for: Evolution of Hominin Polyunsaturated Fatty Acid Metabolism: From Africa to the New World
Source: Genome Biol Evol. 2019 Apr 3;11(5):1417–30. doi: 10.1093/gbe/evz071 (PMC6514828; doi:10.1093/gbe/evz071)
Supplement: Supplementary_Material_evz071 [file supplementary_material_evz071.zip › Supplementary_Tables_and_Figures.pdf]

Figure S1.

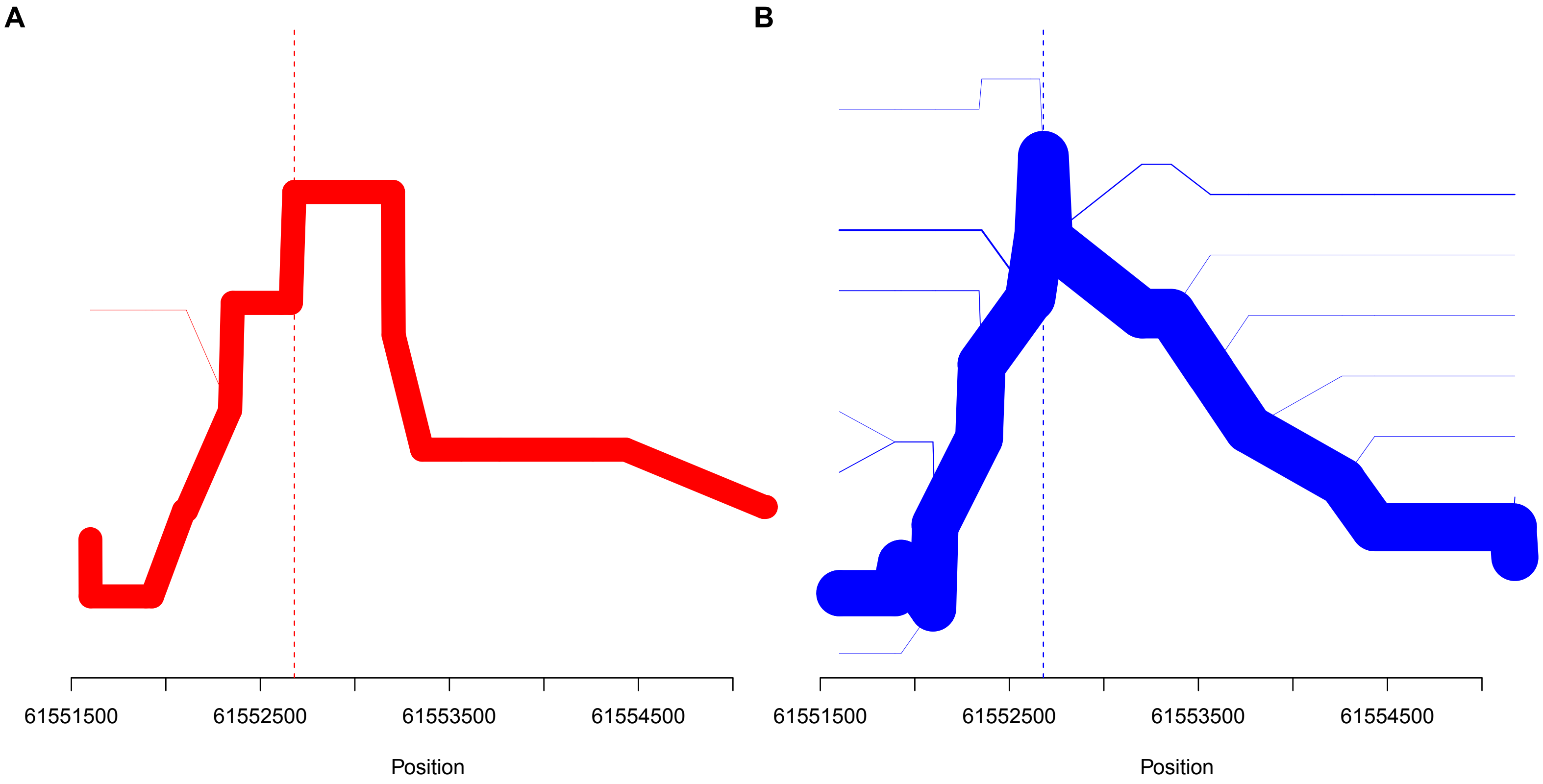
A
B
61551500
61552500
61553500
61554500
Position
61551500
61552500
61553500
61554500
Position

Table S1.

Table S1. FADS Haplotype SNPs in the low coverage dataset.

SNP#	Position
1	chr11:61543499
2	chr11:61543961
3	chr11:61546592
4	chr11:61546888
5	chr11:61547237
6	chr11:61549025
7	chr11:61549458
8	chr11:61551356
9	chr11:61551927
10	chr11:61552680
11	chr11:61557803
12	chr11:61557826
13	chr11:61560081
14	chr11:61564299
15	chr11:61565908
16	chr11:61567753
17	chr11:61569306
18	chr11:61569830
19	chr11:61570783
20	chr11:61571348
21	chr11:61571382
22	chr11:61571478
23	chr11:61573540
24	chr11:61573684
25	chr11:61575158
26	chr11:61579463
27	chr11:61579760
28	chr11:61580504
29	chr11:61580635
30	chr11:61581368
31	chr11:61581450
32	chr11:61581656
33	chr11:61581764
34	chr11:61582708
35	chr11:61585144
36	chr11:61588305
37	chr11:61589481
38	chr11:61591636

Figure S2.

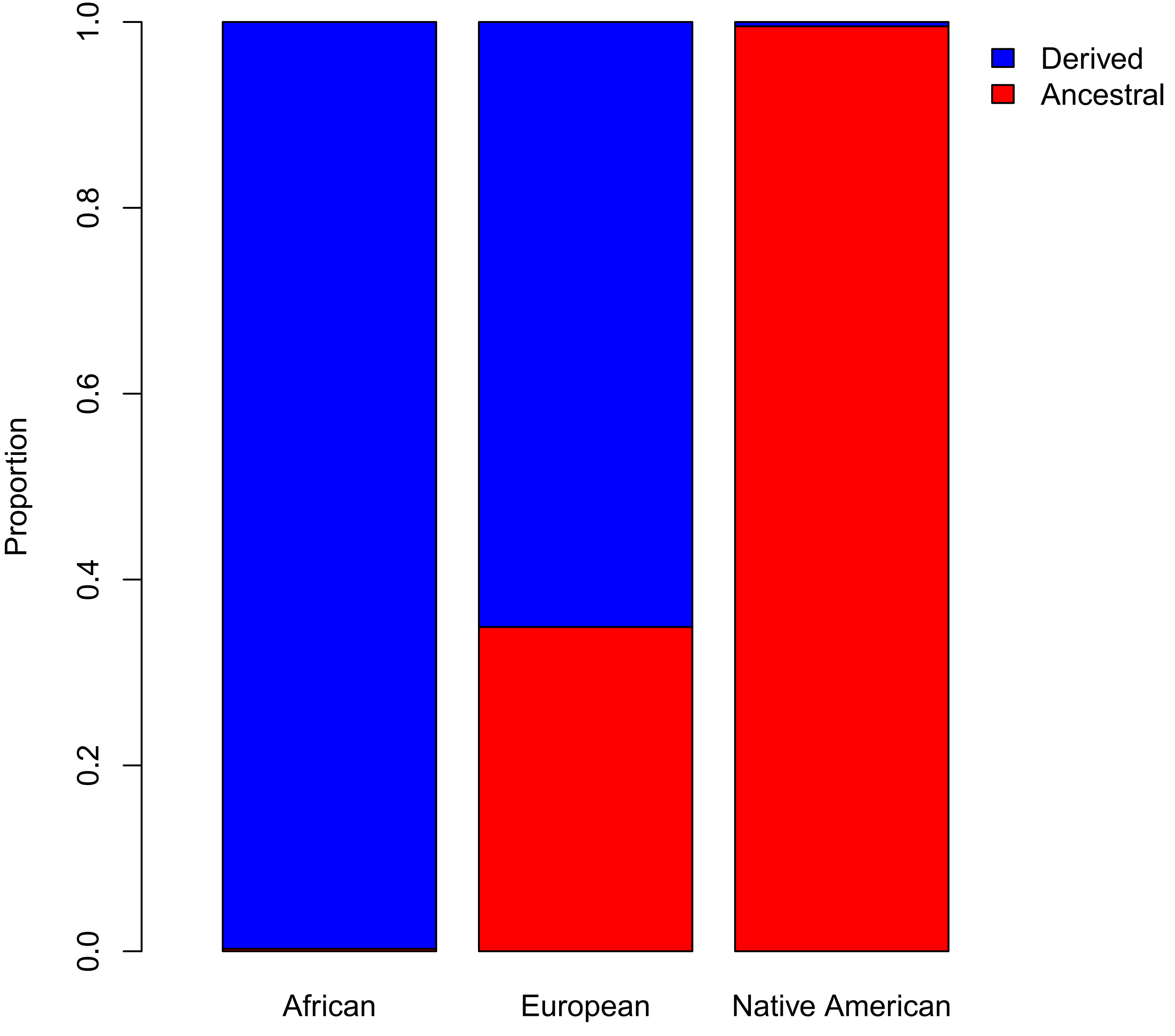
1.0
0.8
0.6
0.4
0.2
0.0
Proportion
African
European
Native American
Derived
Ancestral

Figure S3.

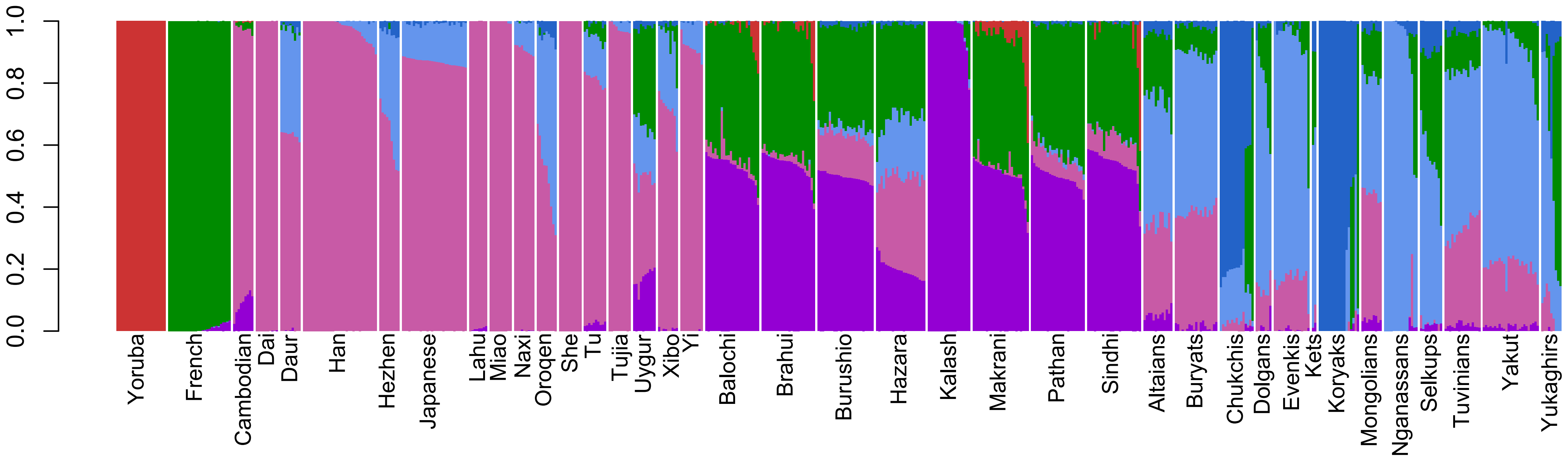

1.0
0.8
0.6
0.4
0.2
0.0
Yoruba
French
Cambodian
Dai
Daur
Han
Hezhen
Japanese
Lahu
Miao
Naxi
Oroqen
She
Tu
Tujia
Uygur
Xibo
Yi
Balochi
Brahui
Burushio
Hazara
Kalash
Makrani
Pathan
Sindhi
Altaians
Buryats
Chukchis
Dolgans
Evenkis
Kets
Koryaks
Mongolians
Nganassans
Selkups
Tuvinians
Yakut
Yukaghirs

Figure S4.

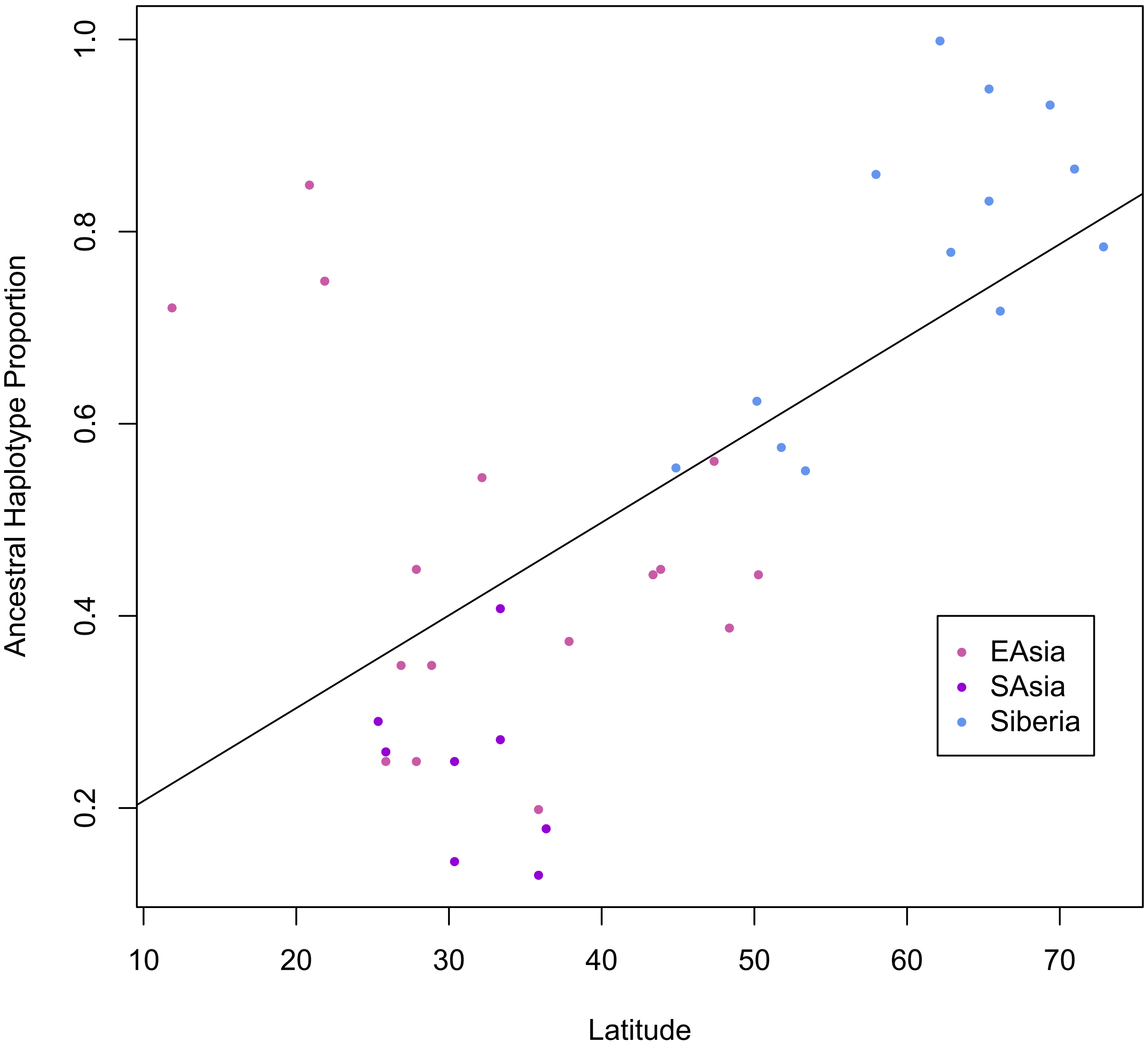
Ancestral Haplotype Proportion
0.2
0.4
0.6
0.8
1.0
10
20
30
40
50
60
70
Latitude
EAsia
SAsia
Siberia

Table S3.

Table S3. Ancestral and Derived haplotype proportions in ancient humans.

Ancient Individual(s)	Region	Age	Sample Size (# of inds)	Ancient Ancestral Proportion	Ancient Derived Proportion
Mota	Africa	4,500	1	0	1
Anzick-1	Americas	13,000	1	1	0
Eskimo	Americas	4,000	1	1	0
Neolithic & Bronze Age	Europe	3,800-8,400	19	0.84	0.16
Mal'ta	Siberia	24,000	1	1	0
Ust'Ishim	Siberia	45,000	1	1	0

Figure S5.

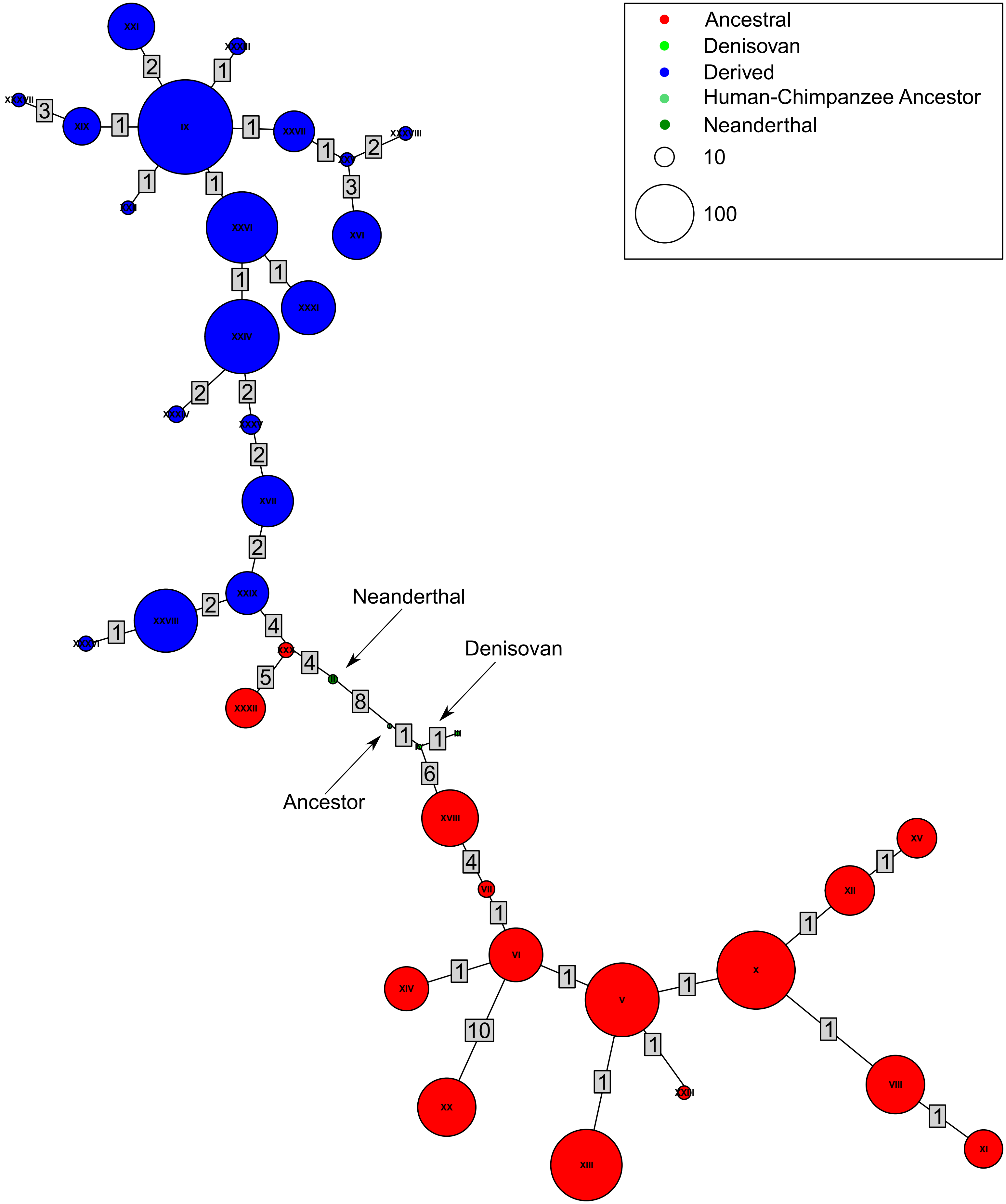

Ancestral
Denisovan
Derived
Human-Chimpanzee Ancestor
Neanderthal
10
100
Neanderthal
Denisovan
Ancestor
IX
XXI
XIX
XXVII
XXVI
XXIV
XXXI
XVI
XVII
XXIX
XXVIII
XXXII
XVIII
VII
VI
V
X
XII
XV
XIV
XX
XIII
VIII
XI

Figure S6.

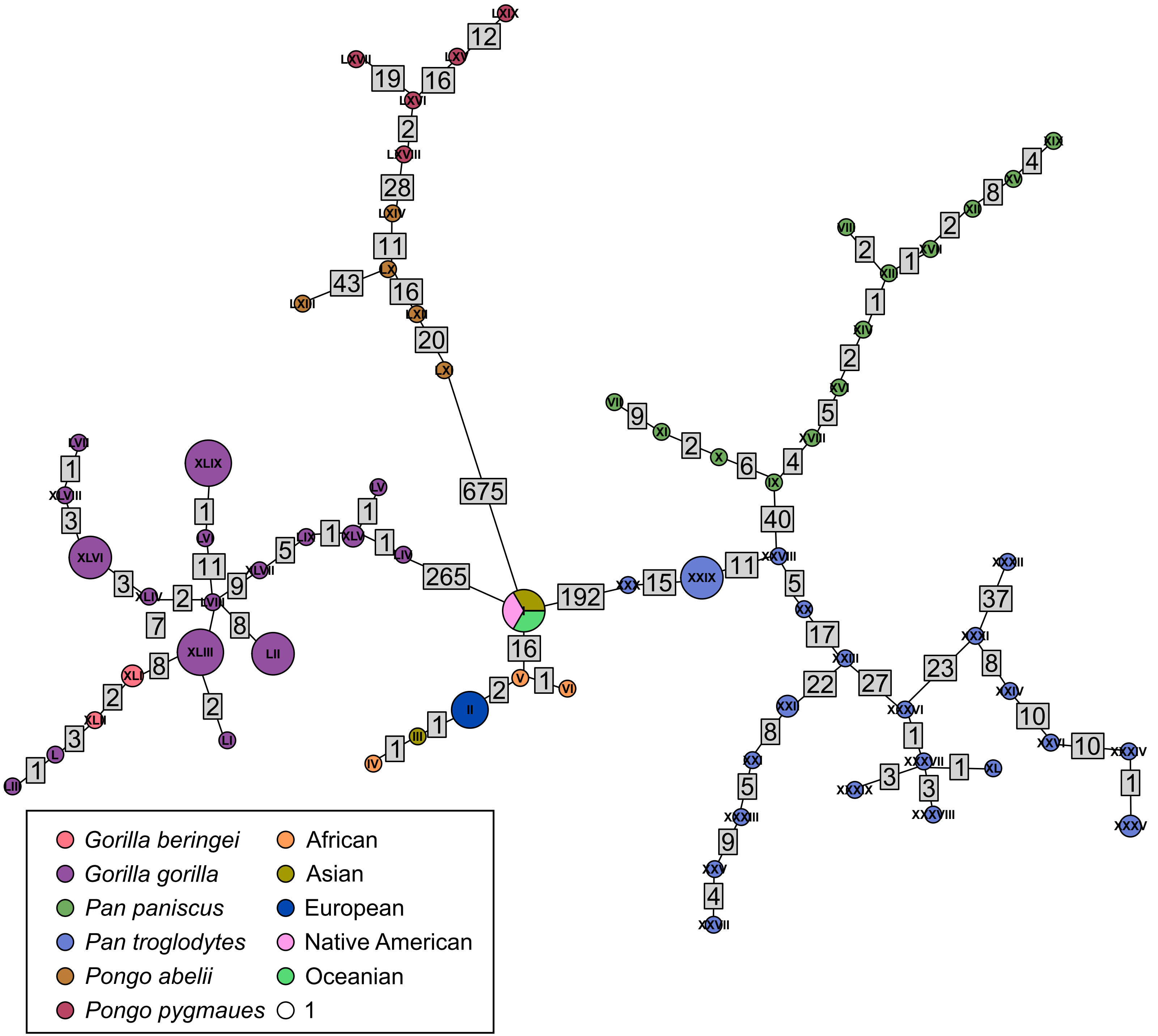
Gorilla beringei
Gorilla gorilla
Pan paniscus
Pan troglodytes
Pongo abelii
Pongo pygmaues
African
Asian
European
Native American
Oceanian
1

Figure S7.

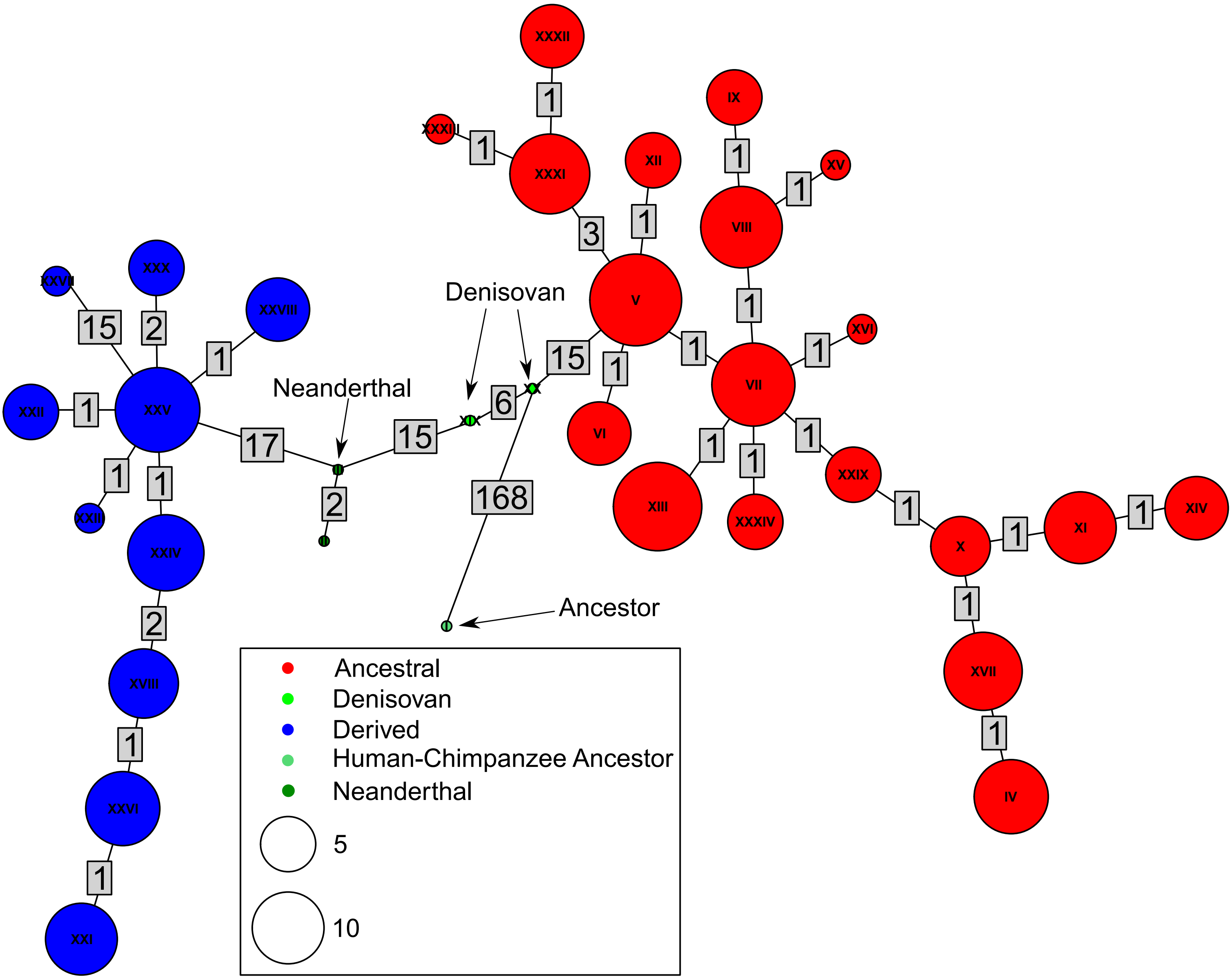
XXXII
XXXIII
XXXI
IX
XII
XV
VIII
V
VII
VI
XVI
XIII
XXXIV
XXIX
X
XI
XIV
XVII
IV
XXX
XXVII
XXVIII
XXII
XXV
XXIII
XXIV
XVIII
XXVI
XXI
Denisovan
Neanderthal
Ancestor
168
Ancestral
Denisovan
Derived
Human-Chimpanzee Ancestor
Neanderthal
5
10

Figure S8.

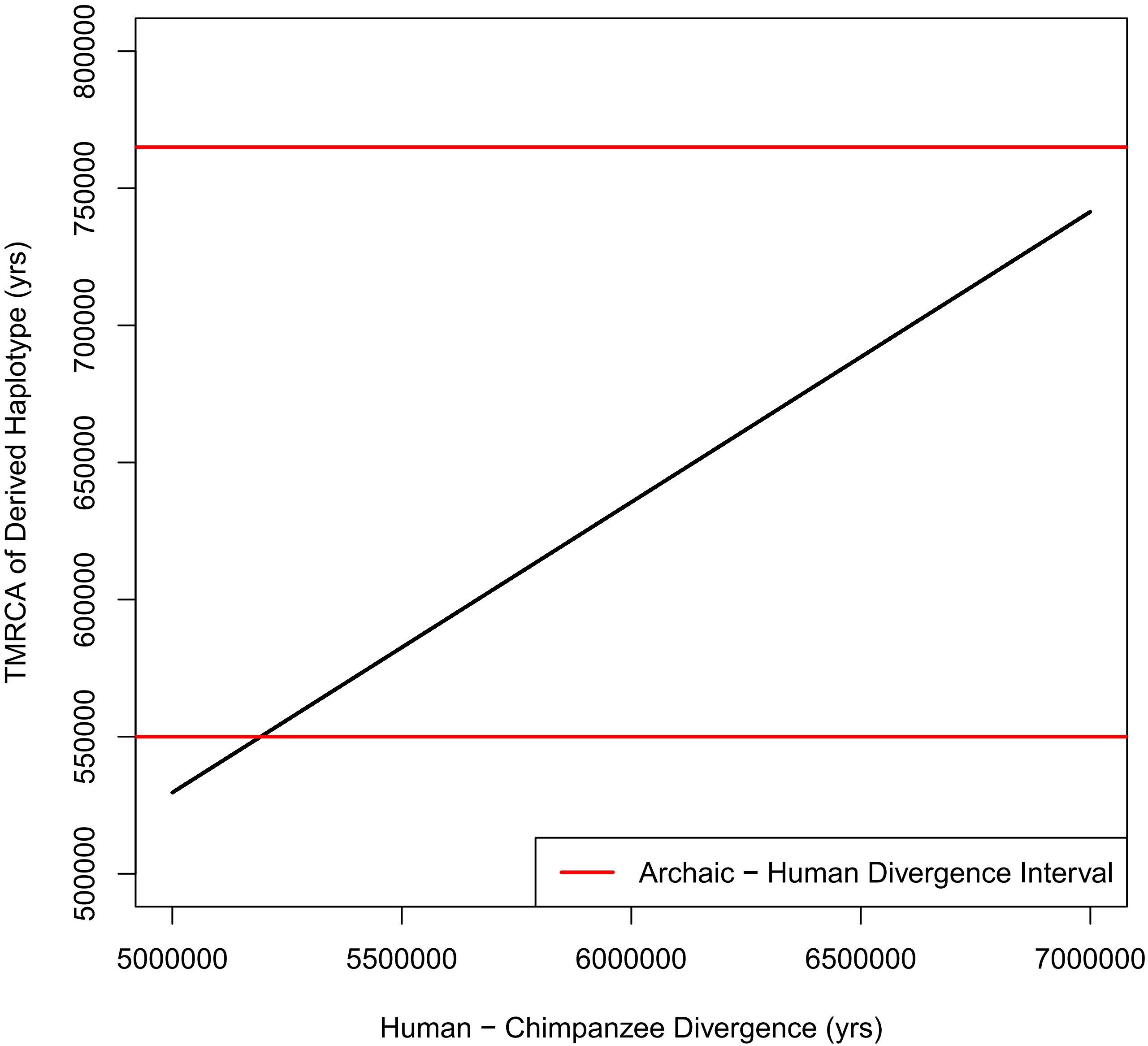

800000
750000
700000
650000
600000
550000
500000
TMRCA of Derived Haplotype (yrs)
Archaic − Human Divergence Interval
5000000
5500000
6000000
6500000
7000000
Human − Chimpanzee Divergence (yrs)
